# Supplementary material for: A Proposal for a Consolidated Structural Model of the CagY Protein of Helicobacter pylori
Source: Int J Mol Sci. 2023 Nov 26;24(23):16781. doi: 10.3390/ijms242316781 (PMC10706595; doi:10.3390/ijms242316781)
Supplement: Supplementary file 1 [file ijms-24-16781-s001.zip › Supplementary_material/Figures/Supplementary_Figures.docx]

**
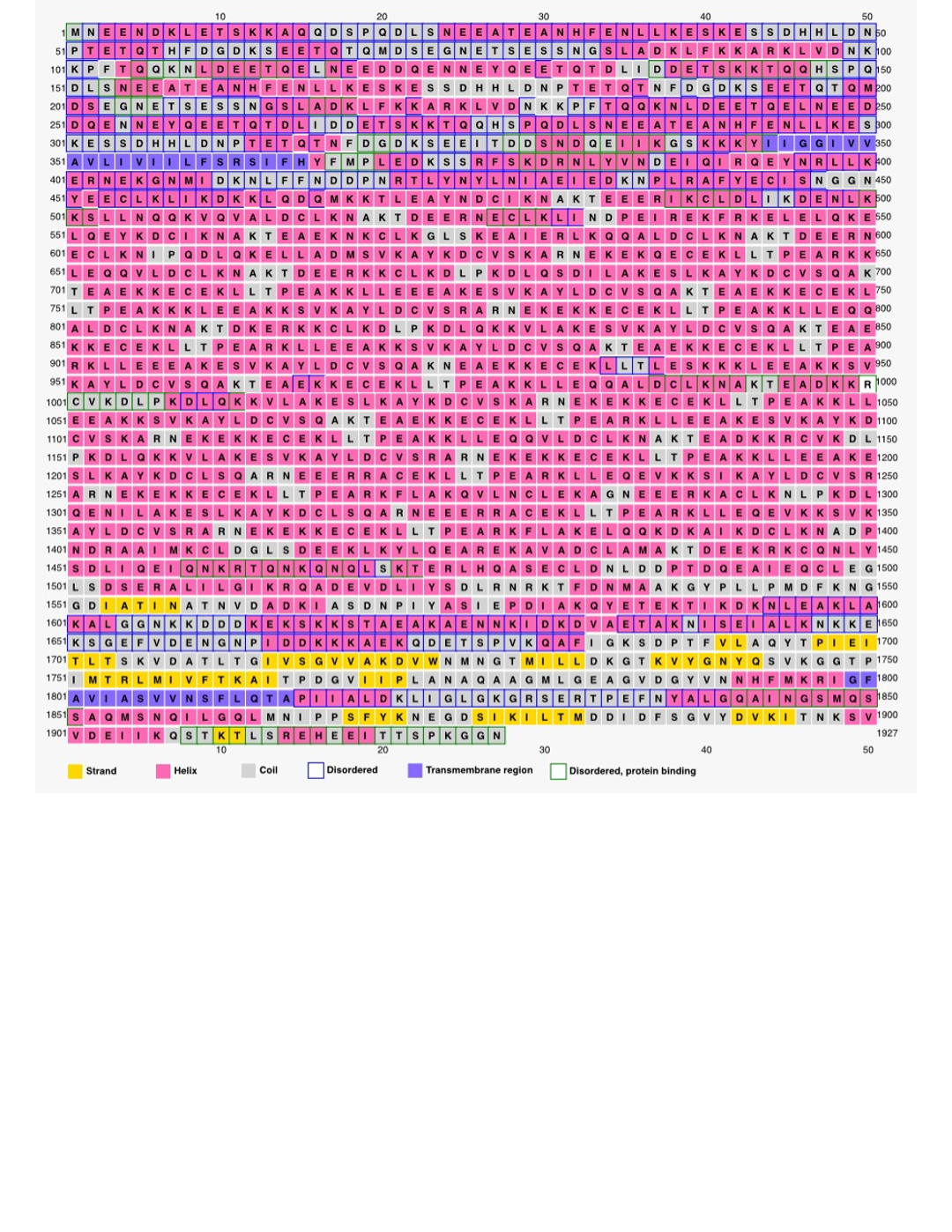
Figure S1.** Secondary structure of CagY. This Scheme shows the predicted secondary structure of CagY protein calculated by PSIPRED server, using the PSIPRED 4.0 and DISOPRED3 options.

**
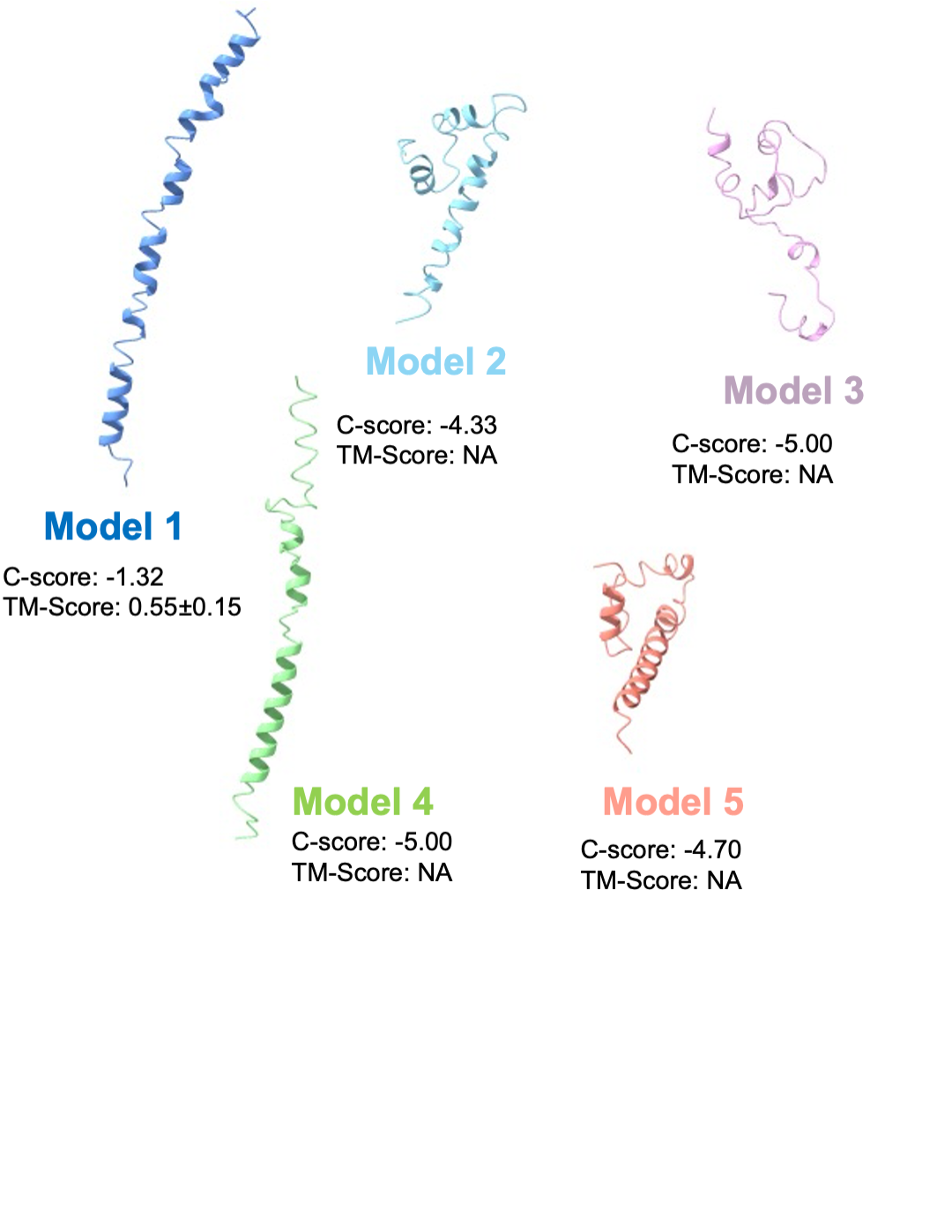
 Figure S2.** The Five I-TASSER models for the missing peptide in the VirB10 region of CagY. This sequence corresponds to the peptide connecting the 6ODI and 6X6J structure regions. Models include their C-score and TM-score values. The best model corresponds to that with the higher C-Score.


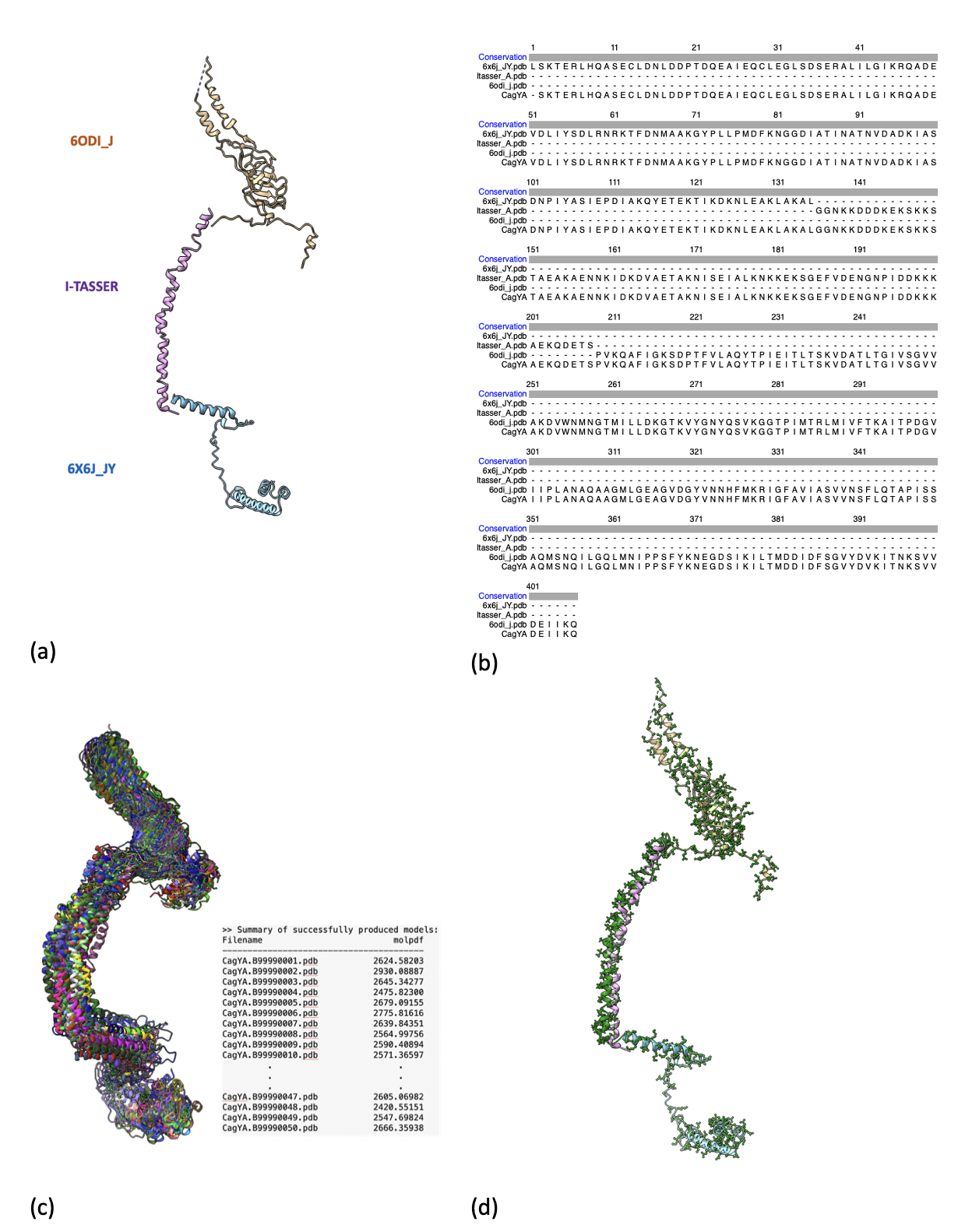


**Figure S3.** Multi-template alignment with Modeller. (a) The I-TASSER predicted model is aligned with 6ODI_J and 6X6J_JY chains. (b) A multi-template alignment with the sequences from the three structures and the CagY sequence that spans those regions is created. (c) A Multi-template modeling script from Modeller is used for building 50 structural models, each having its own Modeller objective function (molpdf), which is a measure of the potential energy of each model. (d) The model with the best fit with 6ODI_J and 6X6J_JY chains and minor molpdf (most stable) was selected.

**
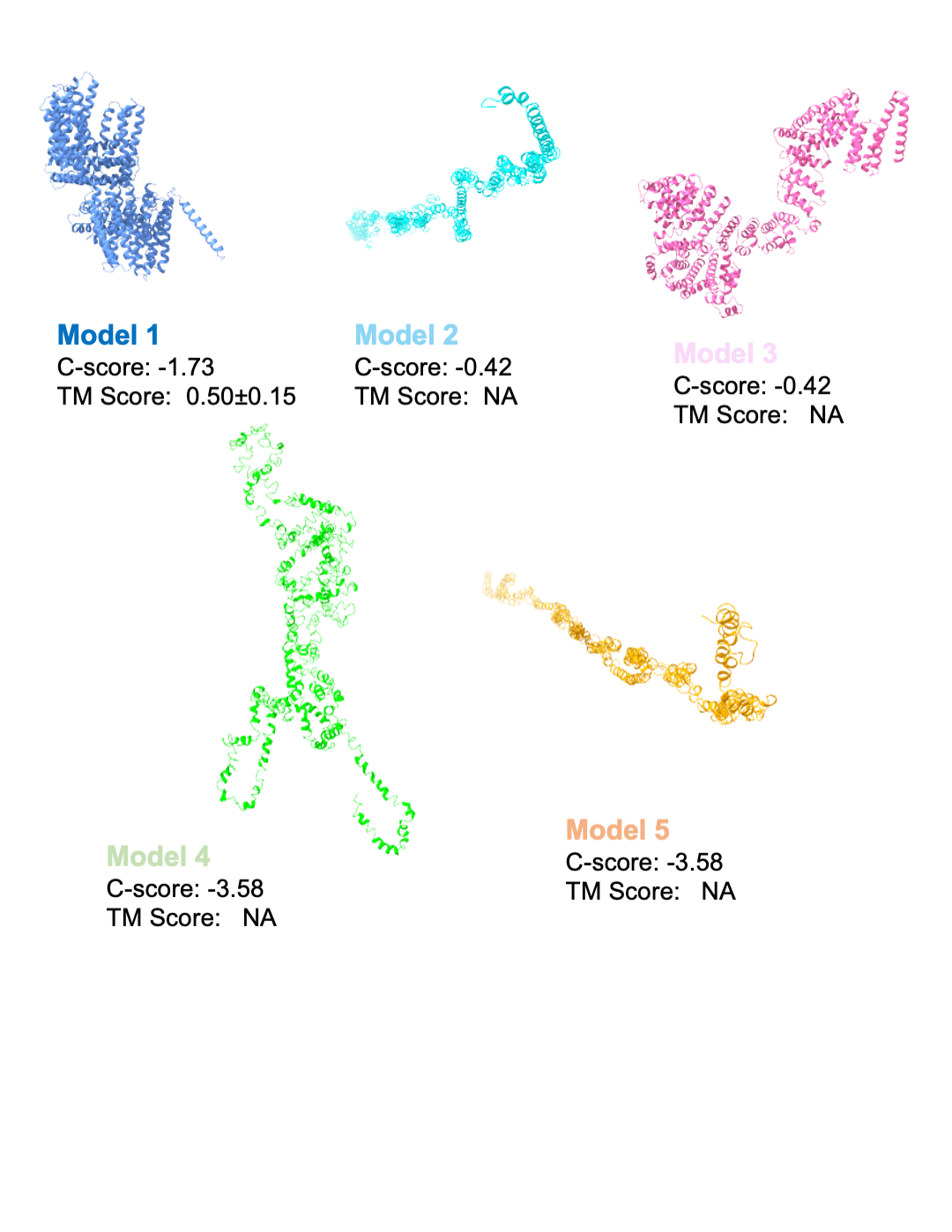
**

**Figure S4.** Five structure models of the MRR of CagY by I-TASSER. Models include their C-score and TM-score values. The best model corresponds to that with the higher C-Score.

**
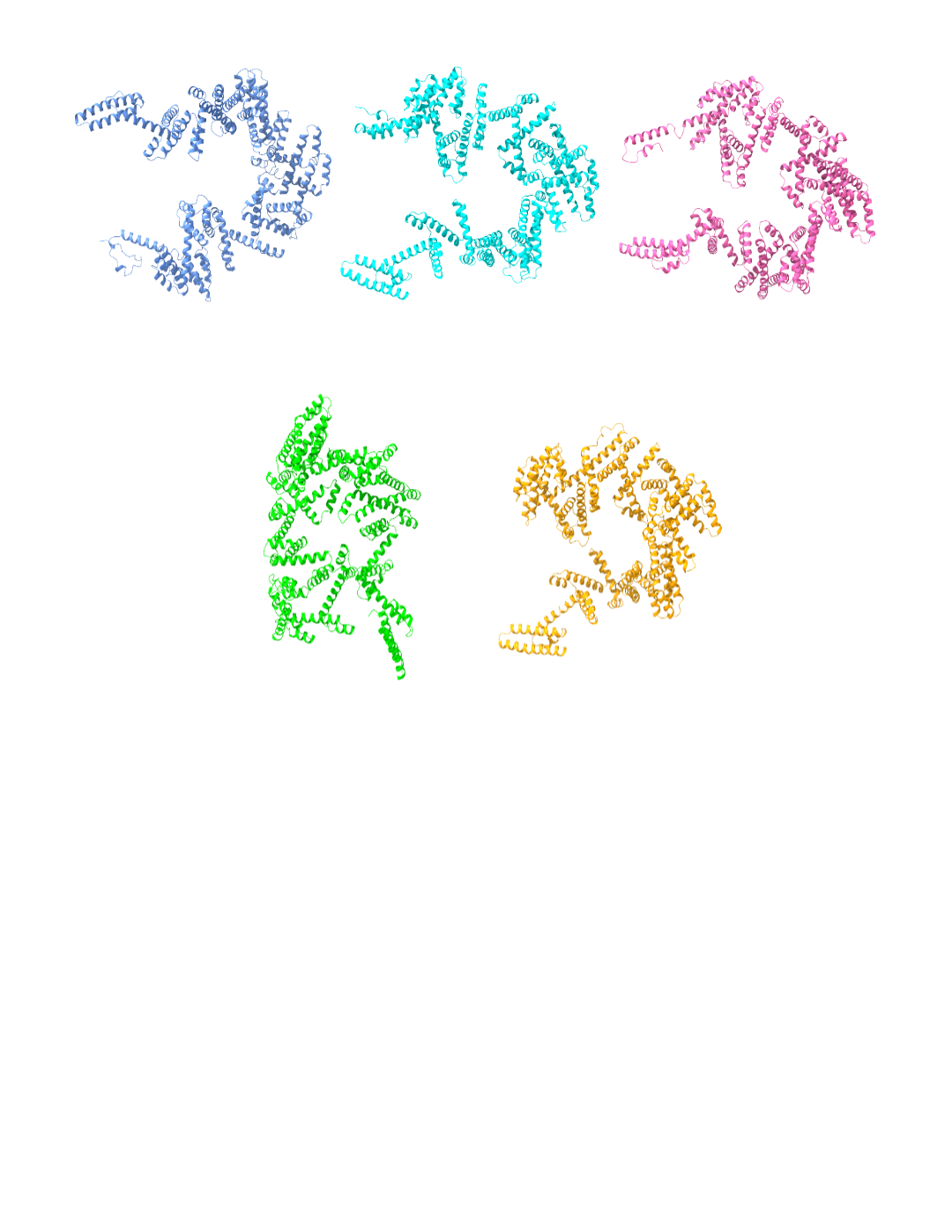
Figure S5.** Five structure models of the MRR of CagY by Robetta. The first model corresponded to the more stable model. No additional data available.

**
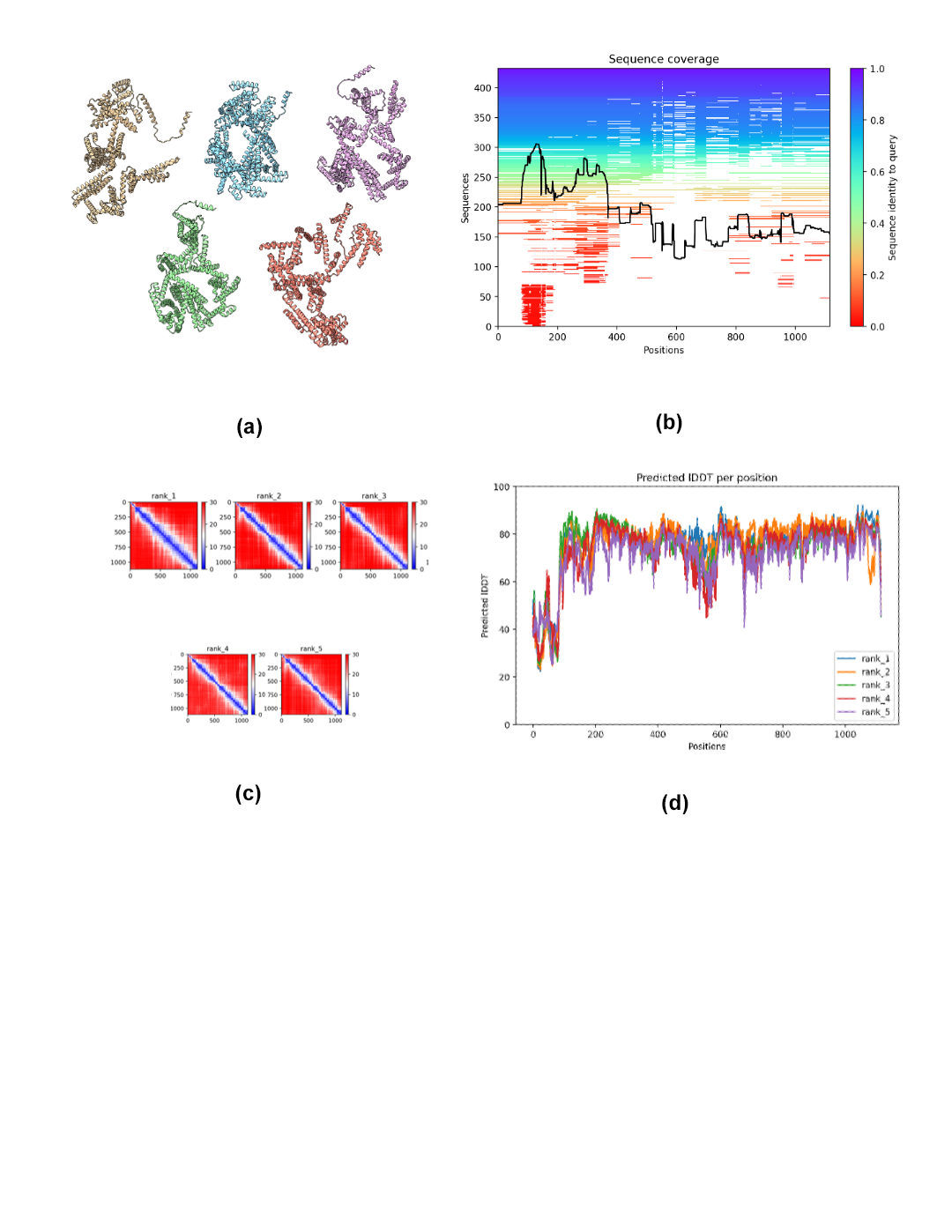
Figure S6.** Structure prediction of MRR of CagY by AlphaFold2/ColabFold. (a) The five models of the structure prediction, ordered by their lDDT predicted ranks. (b) Plot for template coverture in AlphaFold database. (c) Heatmaps of the comparison of the five predicted structures. (d) Predicted lDDT plot of the five models, showing a value higher than 60.

**
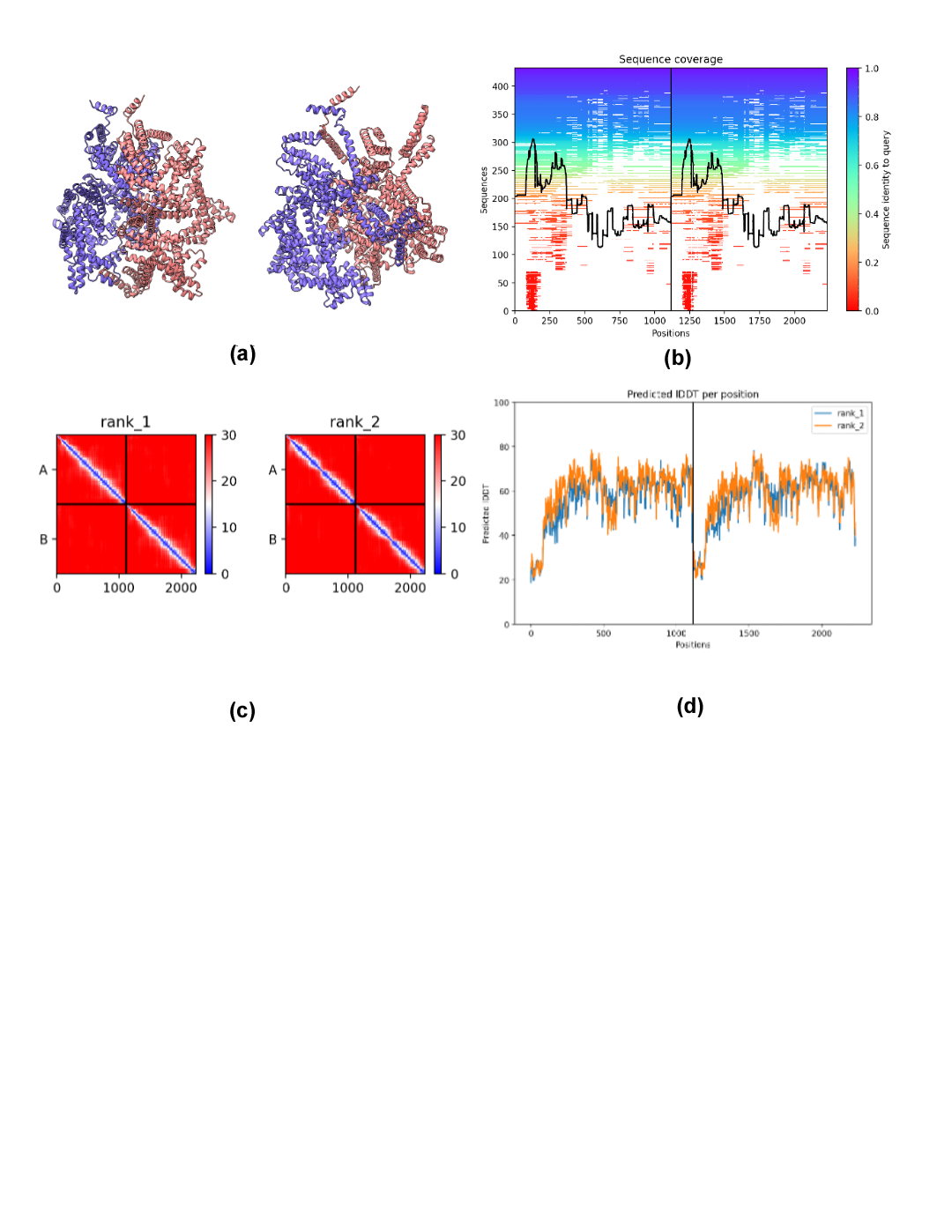
Figure S7.** Structure prediction of the dimer of CagY by AlphaFold2/ColabFold. (a) The two dimer models ordered by their lDDT predicted Rank. (b) Plot for the coverture of templates in the AlphaFold database. (c) Heatmaps for the comparison of the two predicted dimer structures. (d) Predicted IDDT plot of the two dimer predictions, showing values higher than 60.

**
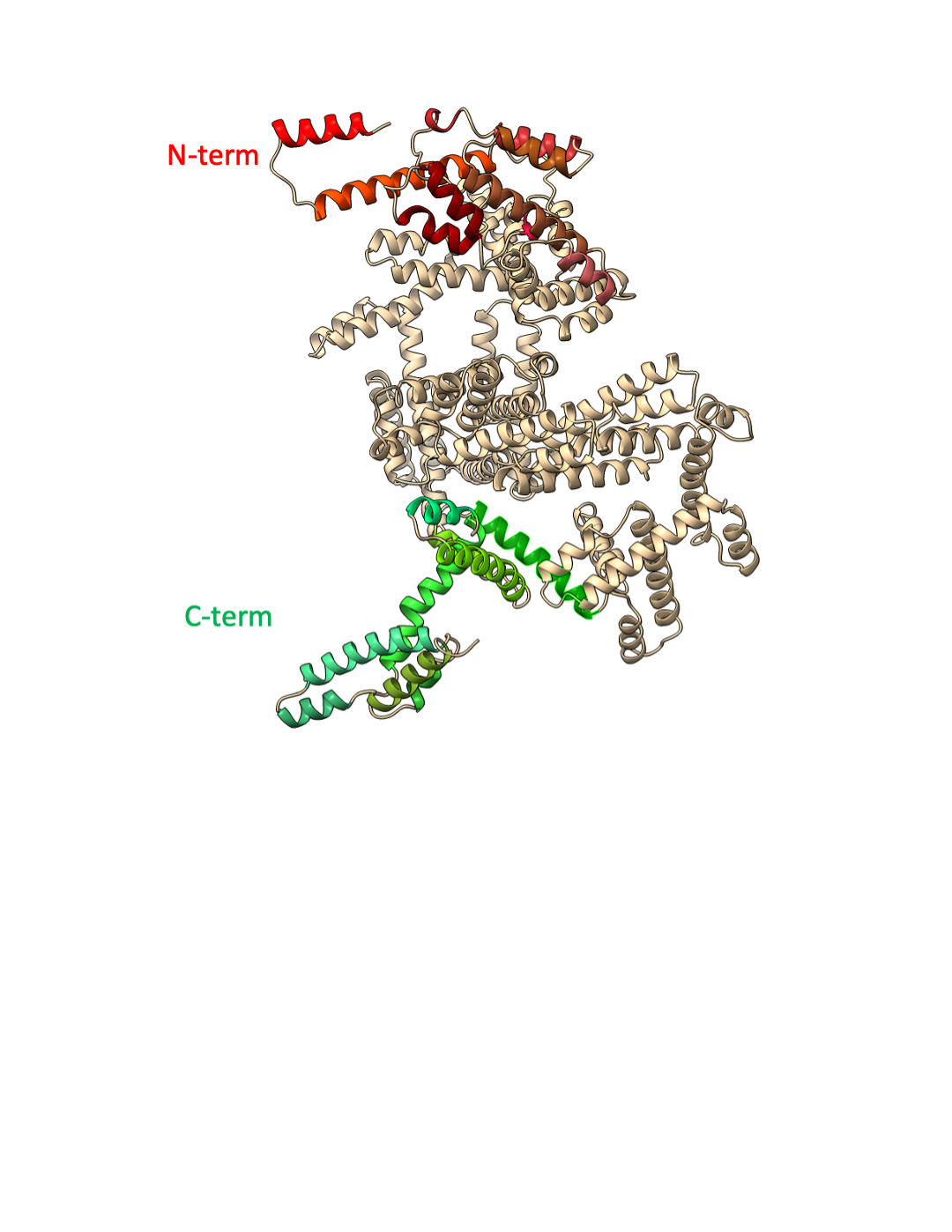
Figure S8. Structure of the MRR from the trimer assembly** optimized by EMD. The regions close to extremes of the protein are highlighted in colors green (C-term) and red (N-term) to orientate the model with respect to the remaining protein structure.

**
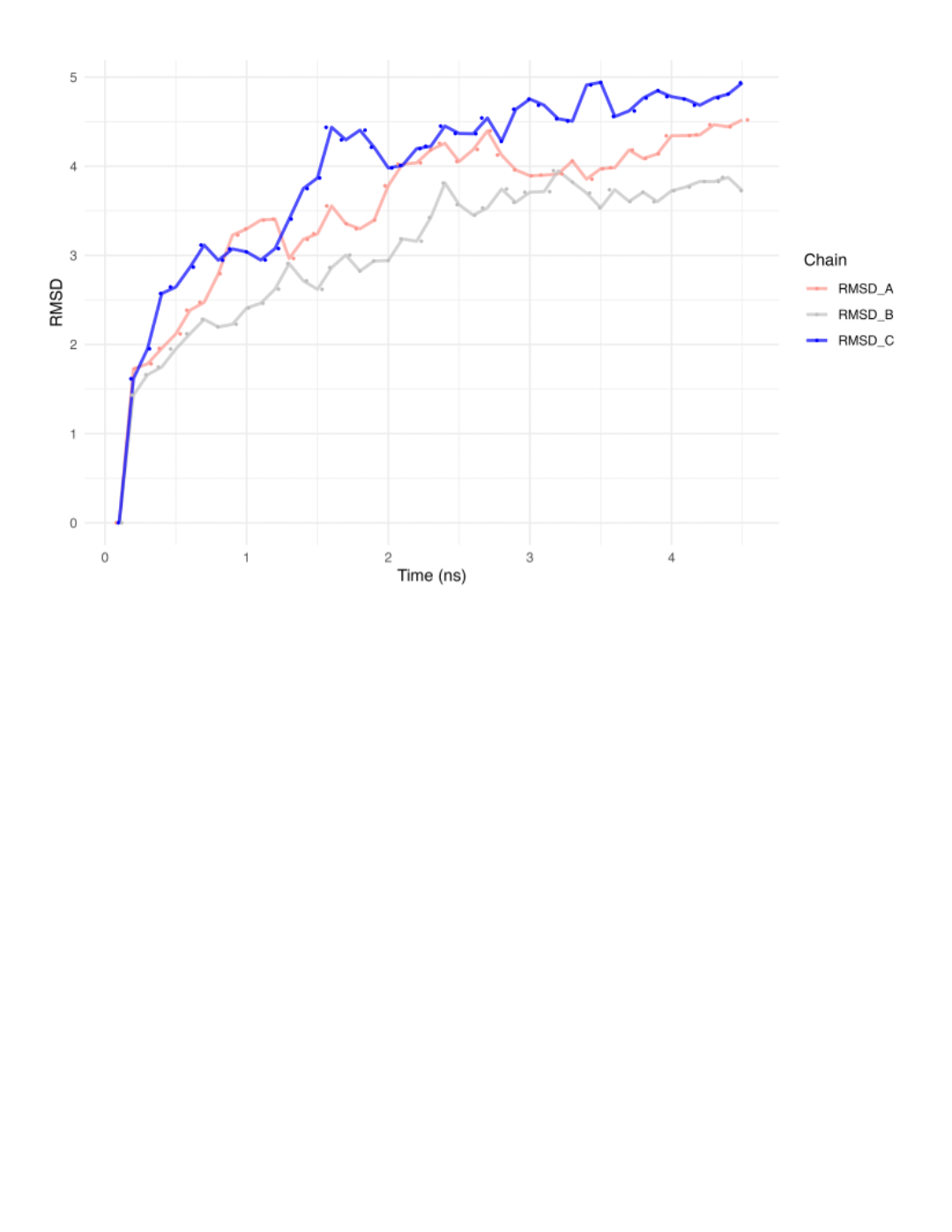
Figure S9. RMSD values of the second EMD of CagY** optimized trimer. A second EMD was run with a trimer build by replacing all the chains with the optimized ones from the previous EMD. The behavior of the dynamics was similar to the previous, with the central chain having less RMSD variation.

**
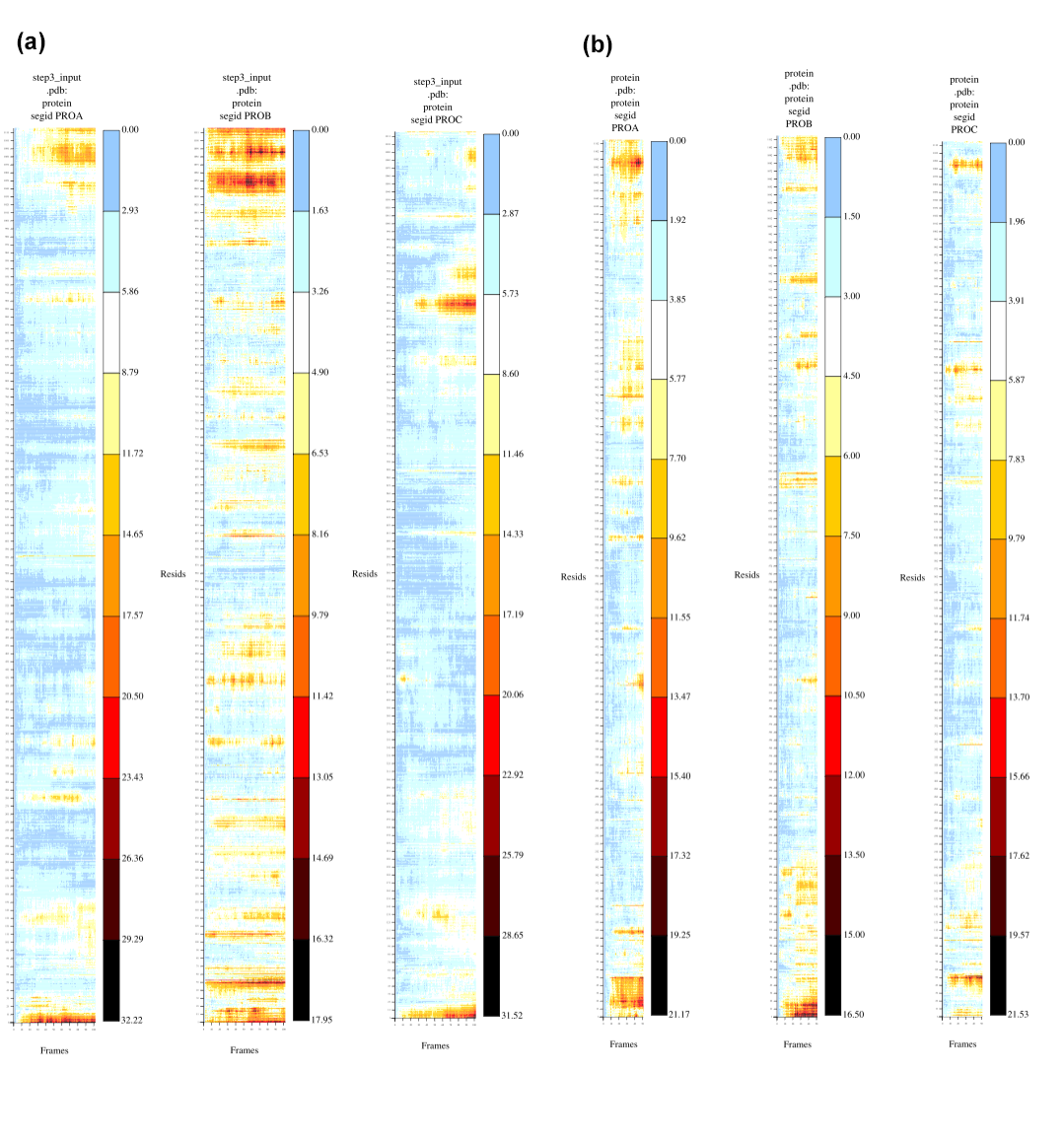
Figure S10.** Heatmaps for the RMSD values per residue/chain in EMD simulations**.** (a) Heatmap for the RMSD values per residue in the first EMD for trimer built from dimers calculated by deep learning. In these plots, the frames correspond to simulation time. The color scale is shown to the right of each heatmap (blue: lower structural variation, red: higher structural variation); PROA, PROB, and PROC refer to the chains of the trimer. (b) Heatmap for a 4.8 ns EMD for a trimer built replacing all three chains with the optimized central chain of the previous EMD. Despite a shorter simulation time for the second EMD, this shows fewer structural internal fluctuations than the first simulation (compare the plots for PROB). Interestingly, structural fluctuations were generally low for chain C (PROC) in both simulations.

**
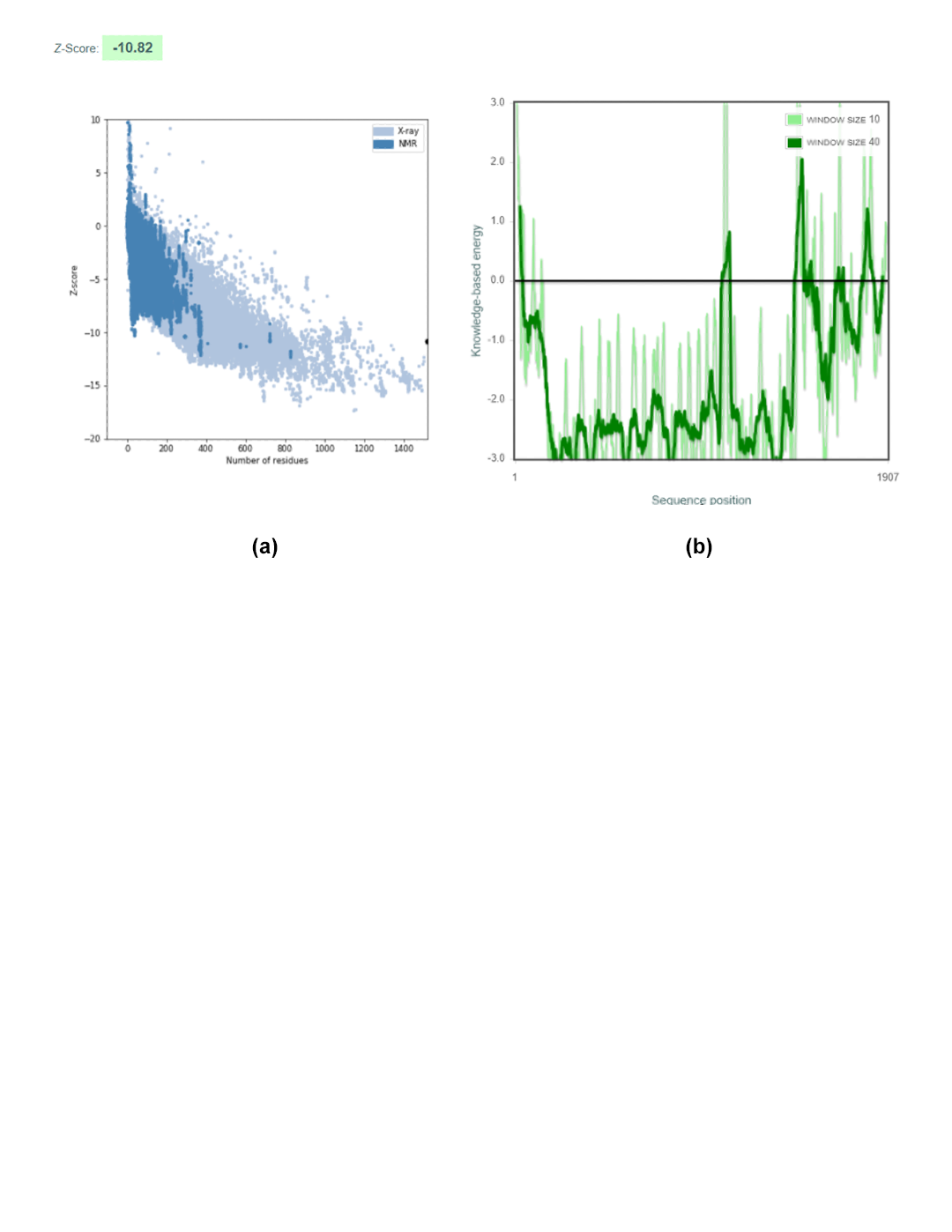
Figure S11.** Validation results of CagY model by the ProSA server. a) The Z score obtained (-10.82) is within the ranges that are obtained in X-ray and Nuclear Magnetic Resonance experiments. However, it is essential to mention that there are few structures reported close to such length. b) The energy graph shows the quality of the local model by plotting energies as a function of the position in the amino acid sequence. The excess of regions with positive energies may be related to modeling errors, and negative energies are related to higher stability and proper modeling.

**
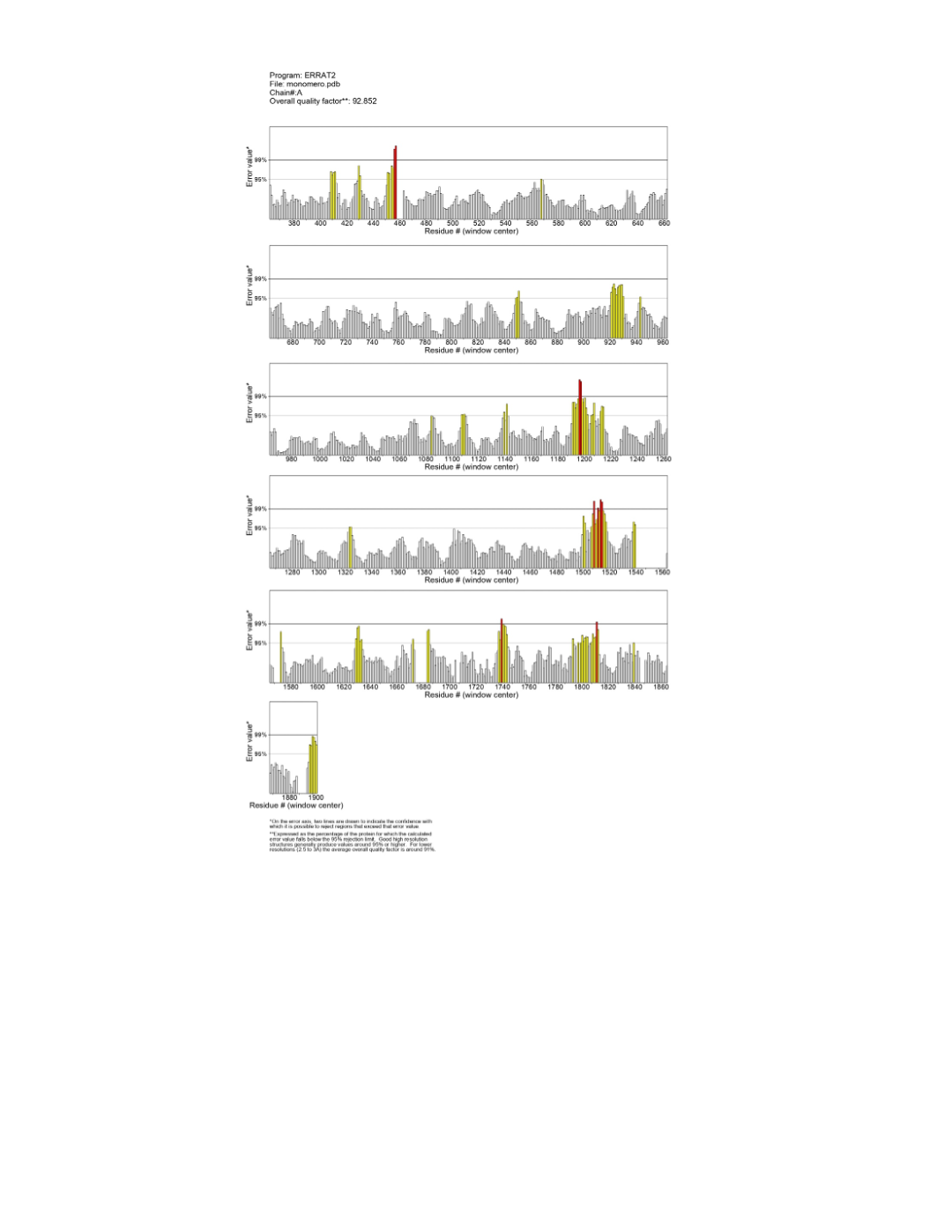
Figure S12.** Validation plot of ERRAT server. The six subgraphs show the sequence of the CagY protein with a 9-residue sliding window, where peaks with errors up to 99% and 95% are highlighted. An overall value of 92.85 is considered acceptable according to the ERRAT algorithm.

**
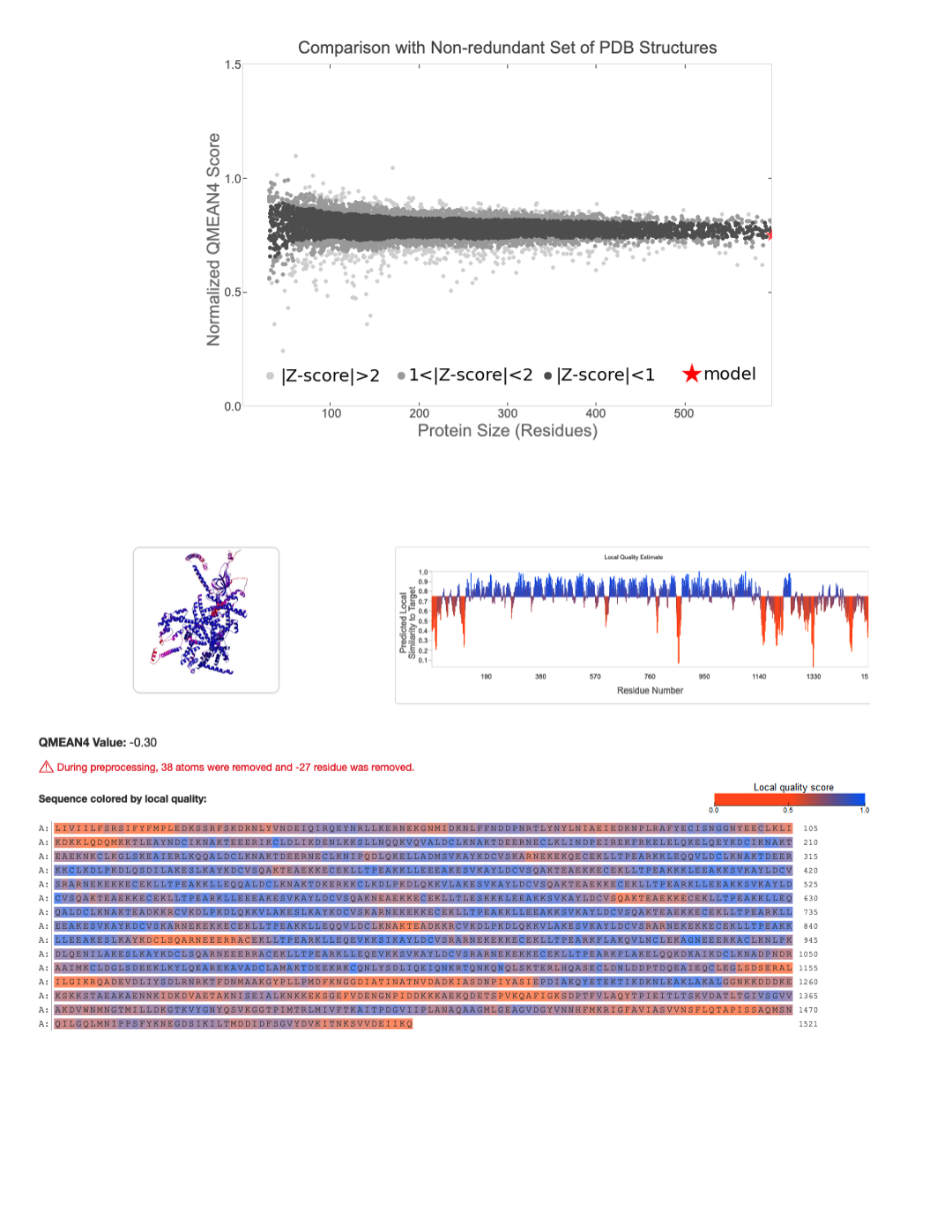
Figure S13.** QMEAN4 validation plot. The QMEAN4 estimates the absolute overall quality (global) and per residue (local) for a structure model. The score is in the range [0,1] and is transformed into a Z-score that relates to the agreement of the model with high-resolution X-ray structures. The calculated score (identified with a red star) is within the observed values, and the Z-score is acceptable. Quality per residue is relatively high for most parts of the modeled structure.

**
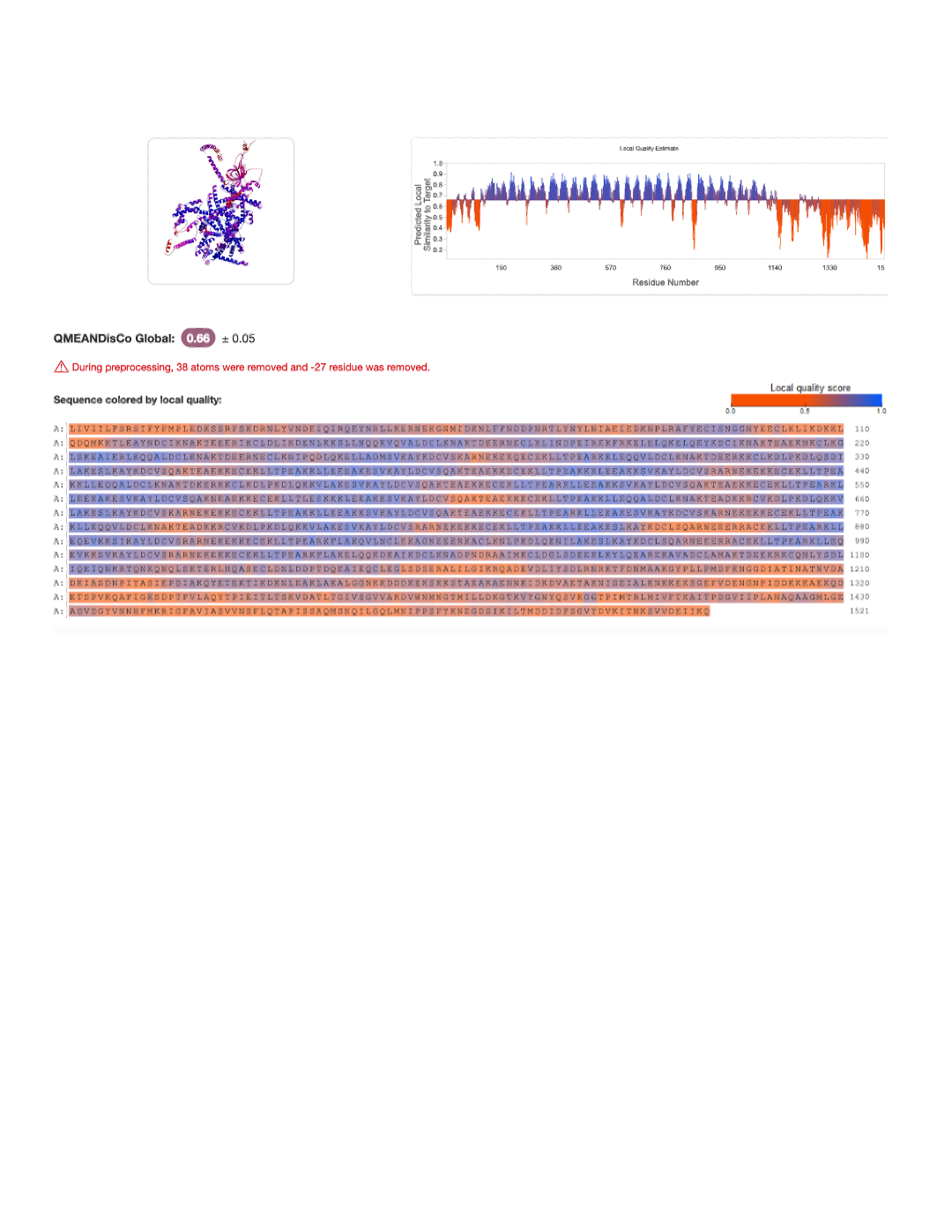
Figure S14.** QMEANDiscCo validation plot. The QMEANDisCo provides absolute quality estimates of the complete model (global) and per residue (local), considering pairwise residue-residue distances from homologous proteins. The overall score is acceptable, and the regions colored in purple correspond to the most confident values.

**
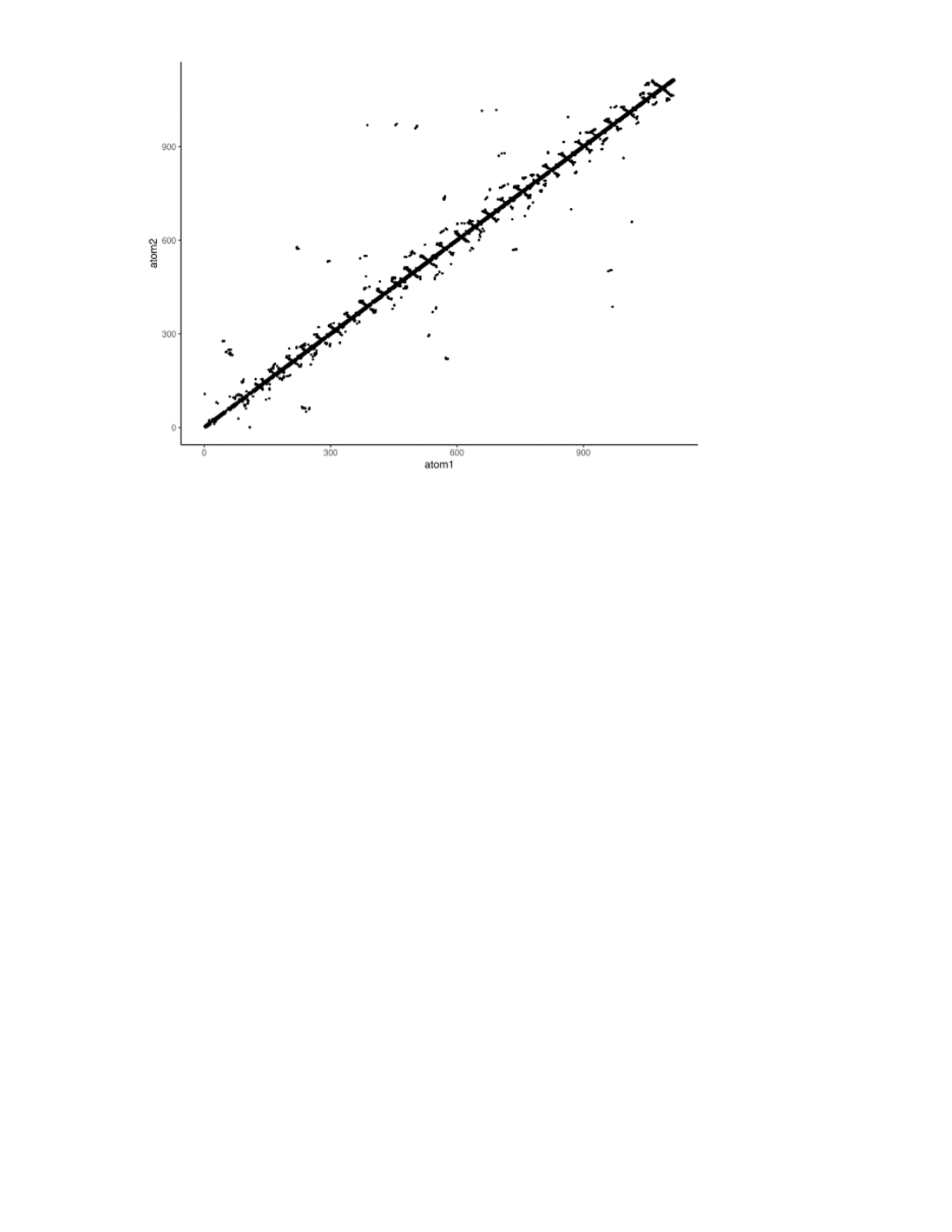
Figure S15.** A plot of residue contacts of the CagY modeled structure. The residue contacts were calculated with an allowed overlap of -0.4 Å and an H-bond overlap reduction of 0.4 Å. The contacts between atoms separated by four bonds or less were ignored. Only intra-molecule contacts were evaluated. A total of 10,404 contacts were estimated, most of them along the main chain. Only one invalid contact (clash) was detected between Glu 825 and Lys 828 with an overlap of 0.603 Å and distance of 2.097 Å. Calculation was conducted with ChimeraX.

**
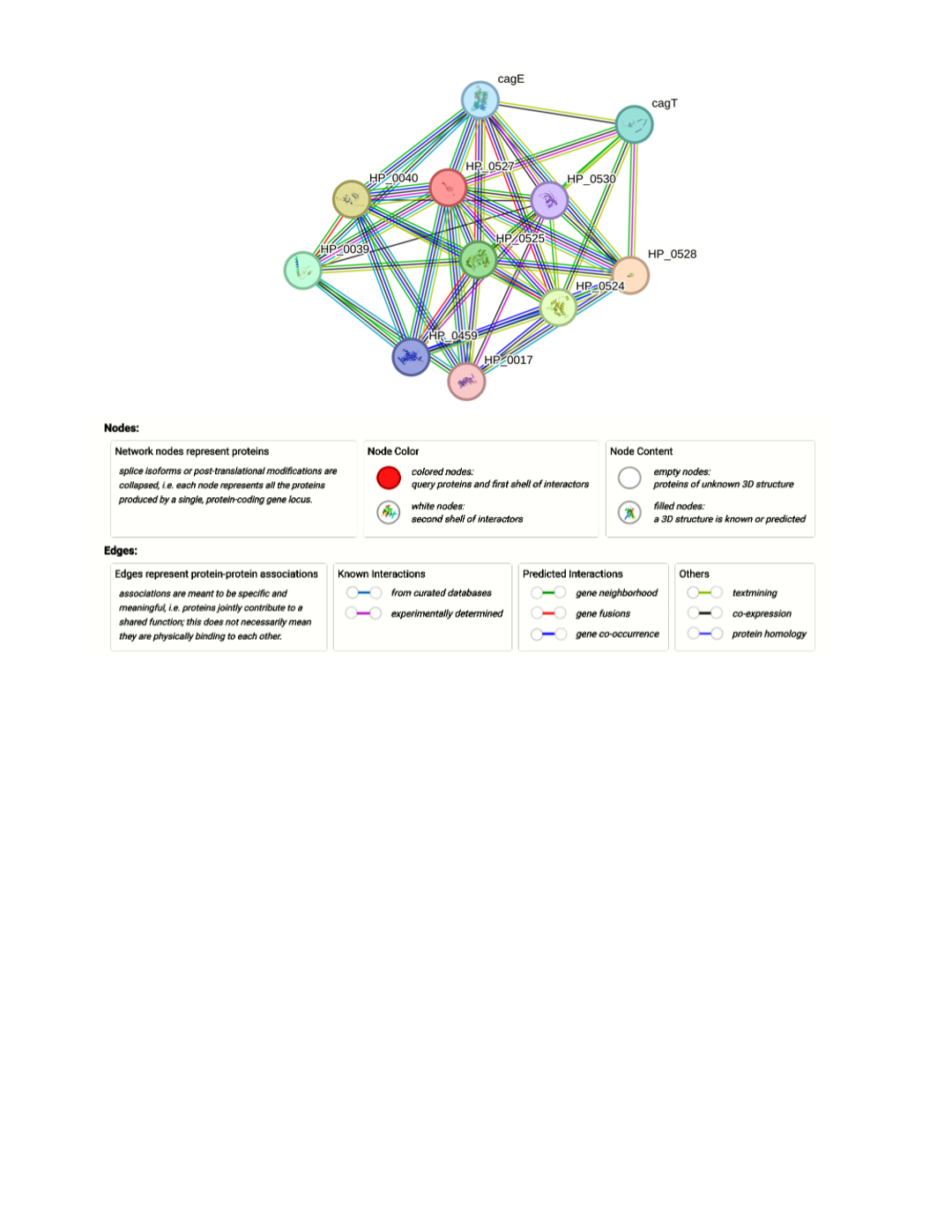
Figure S16. STRING network of CagY protein.** The figure shows the analysis of the known CagY interactions provided by the STRING server*.*
