# Supplementary material for: A Proposal for a Consolidated Structural Model of the CagY Protein of Helicobacter pylori
Source: Int J Mol Sci. 2023 Nov 26;24(23):16781. doi: 10.3390/ijms242316781 (PMC10706595; doi:10.3390/ijms242316781)
Supplement: Supplementary file 1 [file ijms-24-16781-s001.zip › Supplementary_material/Tables/Supplementary_Tables.docx]

**Supplementary Tables**

**Table S1.- STRING network of CagY protein.**

| #node1 | node2 | node1 string_id | node2 string_id | Neighborhood on chromosome | Gene fusion | Phylogenetic cooccurrence | homology | coexpression | Experimentally determined interaction | databaseannotated | Automated textmining | Combined score |
| --- | --- | --- | --- | --- | --- | --- | --- | --- | --- | --- | --- | --- |
| HP_0017 | HP_0039 | 85962.HP_0017 | 85962.HP_0039 | 0.043 | 0 | 0 | 0 | 0.449 | 0 | 0.141 | 0 | 0.507 |
| HP_0017 | HP_0530 | 85962.HP_0017 | 85962.HP_0530 | 0 | 0 | 0 | 0 | 0.405 | 0.318 | 0 | 0 | 0.577 |
| HP_0017 | HP_0528 | 85962.HP_0017 | 85962.HP_0528 | 0 | 0 | 0.325 | 0 | 0.449 | 0 | 0.141 | 0 | 0.652 |
| HP_0017 | HP_0524 | 85962.HP_0017 | 85962.HP_0524 | 0 | 0 | 0.775 | 0 | 0.053 | 0 | 0 | 0.041 | 0.777 |
| HP_0017 | HP_0525 | 85962.HP_0017 | 85962.HP_0525 | 0 | 0 | 0.724 | 0 | 0.346 | 0 | 0 | 0.041 | 0.811 |
| HP_0017 | HP_0040 | 85962.HP_0017 | 85962.HP_0040 | 0.043 | 0 | 0.774 | 0 | 0.449 | 0 | 0.141 | 0 | 0.883 |
| HP_0017 | HP_0527 | 85962.HP_0017 | 85962.HP_0527 | 0.084 | 0 | 0.775 | 0 | 0.597 | 0 | 0.167 | 0.092 | 0.925 |
| HP_0039 | HP_0530 | 85962.HP_0039 | 85962.HP_0530 | 0 | 0 | 0 | 0 | 0.472 | 0 | 0 | 0 | 0.472 |
| HP_0039 | HP_0525 | 85962.HP_0039 | 85962.HP_0525 | 0.042 | 0 | 0 | 0 | 0.476 | 0 | 0 | 0.045 | 0.478 |
| HP_0039 | HP_0459 | 85962.HP_0039 | 85962.HP_0459 | 0.043 | 0 | 0 | 0 | 0.449 | 0 | 0.141 | 0 | 0.507 |
| HP_0039 | cagE | 85962.HP_0039 | 85962.HP_0544 | 0.043 | 0 | 0 | 0 | 0.449 | 0 | 0.141 | 0 | 0.507 |
| HP_0039 | HP_0040 | 85962.HP_0039 | 85962.HP_0040 | 0.773 | 0.862 | 0 | 0 | 0 | 0 | 0 | 0 | 0.967 |
| HP_0039 | HP_0527 | 85962.HP_0039 | 85962.HP_0527 | 0.063 | 0 | 0 | 0 | 0.642 | 0.895 | 0.182 | 0.096 | 0.969 |
| HP_0040 | HP_0530 | 85962.HP_0040 | 85962.HP_0530 | 0 | 0 | 0 | 0 | 0.472 | 0 | 0 | 0 | 0.472 |
| HP_0040 | HP_0525 | 85962.HP_0040 | 85962.HP_0525 | 0.042 | 0 | 0.518 | 0 | 0.476 | 0 | 0 | 0.045 | 0.738 |
| HP_0040 | HP_0524 | 85962.HP_0040 | 85962.HP_0524 | 0.047 | 0 | 0.768 | 0 | 0 | 0 | 0 | 0.045 | 0.77 |
| HP_0040 | cagE | 85962.HP_0040 | 85962.HP_0544 | 0.043 | 0 | 0.765 | 0 | 0.449 | 0 | 0.141 | 0 | 0.879 |
| HP_0040 | HP_0459 | 85962.HP_0040 | 85962.HP_0459 | 0.043 | 0 | 0.775 | 0 | 0.449 | 0 | 0.141 | 0 | 0.884 |
| HP_0040 | HP_0527 | 85962.HP_0040 | 85962.HP_0527 | 0.063 | 0 | 0.761 | 0 | 0.642 | 0.895 | 0.182 | 0.096 | 0.992 |
| HP_0459 | HP_0530 | 85962.HP_0459 | 85962.HP_0530 | 0 | 0 | 0 | 0 | 0.405 | 0.318 | 0 | 0 | 0.576 |
| HP_0459 | HP_0528 | 85962.HP_0459 | 85962.HP_0528 | 0 | 0 | 0.362 | 0 | 0.449 | 0 | 0.141 | 0 | 0.671 |
| HP_0459 | HP_0524 | 85962.HP_0459 | 85962.HP_0524 | 0 | 0 | 0.775 | 0 | 0.053 | 0 | 0 | 0.041 | 0.777 |
| HP_0459 | HP_0525 | 85962.HP_0459 | 85962.HP_0525 | 0 | 0.001 | 0.728 | 0 | 0.346 | 0 | 0 | 0.041 | 0.814 |
| HP_0459 | HP_0527 | 85962.HP_0459 | 85962.HP_0527 | 0.084 | 0 | 0.775 | 0 | 0.596 | 0 | 0.167 | 0.276 | 0.94 |
| HP_0524 | cagT | 85962.HP_0524 | 85962.HP_0532 | 0.352 | 0 | 0 | 0 | 0.046 | 0 | 0 | 0.563 | 0.706 |
| HP_0524 | HP_0530 | 85962.HP_0524 | 85962.HP_0530 | 0.387 | 0 | 0 | 0 | 0 | 0 | 0 | 0.586 | 0.735 |
| HP_0524 | cagE | 85962.HP_0524 | 85962.HP_0544 | 0 | 0.058 | 0.775 | 0 | 0.053 | 0 | 0 | 0.508 | 0.888 |
| HP_0524 | HP_0528 | 85962.HP_0524 | 85962.HP_0528 | 0.568 | 0 | 0.388 | 0 | 0 | 0 | 0 | 0.636 | 0.895 |
| HP_0524 | HP_0525 | 85962.HP_0524 | 85962.HP_0525 | 0.774 | 0.161 | 0.727 | 0 | 0 | 0 | 0 | 0.541 | 0.973 |
| HP_0524 | HP_0527 | 85962.HP_0524 | 85962.HP_0527 | 0.604 | 0 | 0.775 | 0 | 0 | 0.282 | 0 | 0.631 | 0.973 |
| HP_0525 | cagT | 85962.HP_0525 | 85962.HP_0532 | 0.216 | 0 | 0 | 0 | 0 | 0 | 0 | 0.507 | 0.597 |
| HP_0525 | HP_0530 | 85962.HP_0525 | 85962.HP_0530 | 0.387 | 0 | 0 | 0 | 0.363 | 0 | 0 | 0.611 | 0.835 |
| HP_0525 | HP_0528 | 85962.HP_0525 | 85962.HP_0528 | 0.472 | 0 | 0.229 | 0 | 0.476 | 0 | 0 | 0.639 | 0.913 |
| HP_0525 | cagE | 85962.HP_0525 | 85962.HP_0544 | 0 | 0.222 | 0.751 | 0 | 0.346 | 0 | 0 | 0.461 | 0.922 |
| HP_0525 | HP_0527 | 85962.HP_0525 | 85962.HP_0527 | 0.505 | 0 | 0.731 | 0 | 0.495 | 0 | 0 | 0.625 | 0.971 |
| HP_0527 | HP_0530 | 85962.HP_0527 | 85962.HP_0530 | 0.594 | 0 | 0 | 0 | 0.609 | 0 | 0 | 0.61 | 0.933 |
| HP_0527 | cagE | 85962.HP_0527 | 85962.HP_0544 | 0.084 | 0 | 0.775 | 0 | 0.596 | 0 | 0.167 | 0.558 | 0.963 |
| HP_0527 | cagT | 85962.HP_0527 | 85962.HP_0532 | 0.289 | 0 | 0 | 0 | 0.044 | 0.8 | 0 | 0.767 | 0.964 |
| HP_0527 | HP_0528 | 85962.HP_0527 | 85962.HP_0528 | 0.751 | 0 | 0.671 | 0 | 0.642 | 0.978 | 0.182 | 0.814 | 0.999 |
| HP_0528 | cagE | 85962.HP_0528 | 85962.HP_0544 | 0 | 0 | 0.502 | 0 | 0.449 | 0 | 0.141 | 0.534 | 0.875 |
| HP_0528 | HP_0530 | 85962.HP_0528 | 85962.HP_0530 | 0.613 | 0 | 0 | 0 | 0.472 | 0 | 0 | 0.591 | 0.909 |
| HP_0528 | cagT | 85962.HP_0528 | 85962.HP_0532 | 0.447 | 0 | 0 | 0 | 0 | 0.8 | 0 | 0.638 | 0.956 |
| HP_0530 | cagT | 85962.HP_0530 | 85962.HP_0532 | 0.285 | 0 | 0 | 0 | 0 | 0 | 0 | 0.526 | 0.646 |
| HP_0530 | cagE | 85962.HP_0530 | 85962.HP_0544 | 0 | 0 | 0 | 0 | 0.405 | 0.318 | 0 | 0.439 | 0.752 |
| cagE | cagT | 85962.HP_0544 | 85962.HP_0532 | 0 | 0 | 0 | 0 | 0.192 | 0 | 0 | 0.726 | 0.769 |

**Table S2.- Protein Interactions of CagY/Cag7 calculated by STRING server.**

| #term ID | term description | observed gene count | background gene count | strength | false discovery rate | matching proteins in your network (IDs) | matching proteins in your network (labels) |
| --- | --- | --- | --- | --- | --- | --- | --- |
| CL:2854 | Mixed, incl. Bacterial secretion system, and Type IV secretion system, VirB10/TrbI | 11 | 35 | 1.65 | 1.33E-15 | 85962.HP_0017,85962.HP_0039,85962.HP_0040,85962.HP_0459,85962.HP_0524,85962.HP_0525,85962.HP_0527,85962.HP_0528,85962.HP_0530,85962.HP_0532,85962.HP_0544 | HP_0017,HP_0039,HP_0040,HP_0459,HP_0524,HP_0525,HP_0527,HP_0528,HP_0530,cagT,cagE |
| CL:2856 | Bacterial secretion system, and Type IV secretion system, VirB10/TrbI | 10 | 19 | 1.87 | 1.55E-15 | 85962.HP_0017,85962.HP_0039,85962.HP_0040,85962.HP_0524,85962.HP_0525,85962.HP_0527,85962.HP_0528,85962.HP_0530,85962.HP_0532,85962.HP_0544 | HP_0017,HP_0039,HP_0040,HP_0524,HP_0525,HP_0527,HP_0528,HP_0530,cagT,cagE |
| CL:2859 | Bacterial secretion system, and Type IV secretion system, VirB10/TrbI | 7 | 10 | 2 | 8.00E-11 | 85962.HP_0524,85962.HP_0525,85962.HP_0527,85962.HP_0528,85962.HP_0530,85962.HP_0532,85962.HP_0544 | HP_0524,HP_0525,HP_0527,HP_0528,HP_0530,cagT,cagE |
| CL:2869 | Mixed, incl. Protein-exporting ATPase activity, and Type II/IV secretion system protein | 4 | 5 | 2.06 | 8.25E-06 | 85962.HP_0524,85962.HP_0525,85962.HP_0530,85962.HP_0544 | HP_0524,HP_0525,HP_0530,cagE |
| CL:2860 | Mixed, incl. Conjugal transfer protein, and Type IV secretion system, VirB10/TrbI | 3 | 5 | 1.93 | 0.00063 | 85962.HP_0527,85962.HP_0528,85962.HP_0532 | HP_0527,HP_0528,cagT |
| CL:2881 | Mixed, incl. Conjugal transfer protein, and Helicase HerA, central domain | 3 | 5 | 1.93 | 0.00063 | 85962.HP_0017,85962.HP_0039,85962.HP_0040 | HP_0017,HP_0039,HP_0040 |

**Table S3.- Predicted disulfide bonds by PDBsum.**

| 1st cysteine | 2nd cysteine | Type (*) | Chi1 | Chi2 | Chi3 | Chi2p | Chi1p |
| --- | --- | --- | --- | --- | --- | --- | --- |
| A 92 | A 102 | LHS | -179.7 | -88.4 | -73.6 | -53.6 | -64.9 |
| A 125 | A 138 | LHS | -177.3 | -65.0 | -77.7 | -66.9 | -64.7 |
| A 163 | A 176 | LHS | -178.5 | -61.7 | -65.8 | -67.2 | -65.6 |
| A 205 | A 218 | LHS | -176.6 | -67.7 | -78.3 | -62.3 | -61.1 |
| A 237 | A 250 | LHS | -177.0 | -67.7 | -73.0 | -60.8 | -63.2 |
| A 274 | A 287 | LHS | -179.3 | -79.1 | -71.6 | -67.7 | -61.9 |
| A 306 | A 319 | LHS | -178.0 | -71.8 | -68.0 | -85.2 | -61.7 |
| A 343 | A 356 | LHS | -179.6 | -76.7 | -69.1 | -71.9 | -65.4 |
| A 382 | A 395 | LHS | 179.1 | -75.8 | -73.5 | -63.5 | -63.7 |
| A 420 | A 433 | LHS | -179.4 | -85.1 | -64.9 | -78.1 | -63.2 |
| A 452 | A 465 | LHS | -176.6 | -79.1 | -62.5 | -79.9 | -61.0 |
| A 489 | A 502 | LHS | 179.8 | -78.8 | -72.3 | -63.8 | -63.1 |
| A 527 | A 540 | LHS | -178.6 | -65.4 | -73.7 | -71.1 | -63.5 |
| A 566 | A 579 | LHS | -179.9 | -76.8 | -76.5 | -55.8 | -62.3 |
| A 604 | A 617 | LHS | -179.0 | -74.9 | -71.5 | -67.5 | -62.8 |
| A 636 | A 649 | LHS | -177.4 | -70.3 | -69.2 | -77.4 | -61.7 |
| A 673 | A 686 | LHS | -178.5 | -64.1 | -73.7 | -71.6 | -62.9 |
| A 711 | A 724 | LHS | 179.9 | -76.8 | -72.5 | -64.7 | -62.9 |
| A 749 | A 762 | LHS | -178.9 | -72.8 | -71.2 | -77.2 | -61.6 |
| A 781 | A 794 | LHS | -178.3 | -83.7 | -61.3 | -88.6 | -62.7 |
| A 818 | A 831 | LHS | -179.9 | -80.4 | -66.3 | -77.8 | -63.7 |
| A 856 | A 869 | LHS | -177.8 | -78.5 | -71.6 | -61.9 | -62.8 |
| A 895 | A 908 | LHS | -177.8 | -81.1 | -63.3 | -81.3 | -63.2 |
| A 927 | A 940 | LHS | -177.9 | -75.0 | -63.7 | -91.0 | -63.9 |
| A 964 | A 977 | LHS | -178.5 | -66.0 | -75.7 | -74.1 | -62.3 |
| A 1003 | A 1016 | LHS | -179.5 | -76.8 | -75.1 | -59.6 | -63.6 |
| A 1042 | A 1057 | LHS | -174.6 | -87.2 | -59.0 | -79.0 | -63.3 |
| A 1081 | A 1094 | LHS | -175.2 | -84.9 | -53.6 | -78.8 | -66.7 |

(*) The data displayed for each disulphide bond is as follows: The table shows the residue numbers of the two cysteines involved in the disulphide bridge and the type of disulphide. The type of bridge is abbreviated (RHH: right hand hook; SRH: short right hand hook; LHS: left handed spiral; RHS: right handed spiral). Chi1, chi2, chi3, chi2' and chi1' values and the distance between the C-alpha atoms of the residues involved are also recorded.

**Table S4.- Protein Templates used by the I-TASSER server to model the MRR of CagY.**

| PDB Hit | PDB accesion | RMSD | Protein name |
| --- | --- | --- | --- |
| 1 | 7kogB | 0.675 | Lethocerus Myosin II complete coiled-coil domain resolved in its native environment |
| 2 | 3hr2A | 0.256 | Low resolution, molecular envelope structure of type I collagen in situ determined by fiber diffraction. Single type I collagen molecule, post rigid body refinement, 'relaxed' |
| 3 | 1c1gA | 0.193 | CRYSTAL STRUCTURE OF TROPOMYOSIN AT 7 ANGSTROMS RESOLUTION IN THE SPERMINE-INDUCED CRYSTAL FORM |
| 4 | 6yvuB | 0.171 | Condensin complex from S.cerevisiae ATP-free apo non-engaged state |
| 5 | 6f1tX | 0.168 | Cryo-EM structure of two dynein tail domains bound to dynactin and BICDR1 |
| 6 | 6z9lA | 0.163 | Enterococcal PrgA |
| 7 | 1sjjA | 0.161 | Cryo-EM Structure of Chicken Gizzard Smooth Muscle alpha-Actinin |
| 8 | 6jlbA | 0.158 | Crystal structure of lamin A/C fragment and assembly mechanisms of intermediate filaments |
| 9 | 6l5jA | 0.155 | Crystal structure of human rootletin 1108-1317 |

**Table S5.- AP sequences**

| AP model | Sequence |
| --- | --- |
| WT | FLQTAPIIALDKLIGLGKGRSERTPEFNYALGQAINGSMQSSAQMSNQILGQ |
| GS20 | FLQTAPIIALDKLIGLGSGSGSGSGSGSGSGSGSGSGSMQSSAQMSNQILGQ |
| Xc (*) | FLQTAPIIALDKLIGVGSGIVTQQPFESNTSMQSSAQMSNQILGQ |
|  |  |
|  |  |

***(*) Xanthomonas citri***
