# Supplementary material for: A Proposal for a Consolidated Structural Model of the CagY Protein of Helicobacter pylori
Source: Int J Mol Sci. 2023 Nov 26;24(23):16781. doi: 10.3390/ijms242316781 (PMC10706595; doi:10.3390/ijms242316781)
Supplement: Supplementary file 1 [file ijms-24-16781-s001.zip › Supplementary_material/Tables/Supplementary_Tables.pdf]

Supplementary Tables

Table S1.- STRING network of CagY protein.

#node 1	node2	node1 string_id	node2 string_id	Neighborhood on chromosome	Gene fusion	Phylogenetic cooccurrence	homol ogy	coexpress ion	Experimentally determined interaction	databaseanno tated	Automated textmining	Combined score
HP_0017	HP_0039	85962.HP_0017	85962.HP_0039	0.043	0	0	0	0.449	0	0.141	0	0.507
HP_0017	HP_0530	85962.HP_0017	85962.HP_0530	0	0	0	0	0.405	0.318	0	0	0.577
HP_0017	HP_0528	85962.HP_0017	85962.HP_0528	0	0	0.325	0	0.449	0	0.141	0	0.652
HP_0017	HP_0524	85962.HP_0017	85962.HP_0524	0	0	0.775	0	0.053	0	0	0.041	0.777
HP_0017	HP_0525	85962.HP_0017	85962.HP_0525	0	0	0.724	0	0.346	0	0	0.041	0.811
HP_0017	HP_0040	85962.HP_0017	85962.HP_0040	0.043	0	0.774	0	0.449	0	0.141	0	0.883
HP_0017	HP_0527	85962.HP_0017	85962.HP_0527	0.084	0	0.775	0	0.597	0	0.167	0.092	0.925
HP_0039	HP_0530	85962.HP_0039	85962.HP_0530	0	0	0	0	0.472	0	0	0	0.472
HP_0039	HP_0525	85962.HP_0039	85962.HP_0525	0.042	0	0	0	0.476	0	0	0.045	0.478
HP_0039	HP_0459	85962.HP_0039	85962.HP_0459	0.043	0	0	0	0.449	0	0.141	0	0.507
HP_0039	cagE	85962.HP_0039	85962.HP_0544	0.043	0	0	0	0.449	0	0.141	0	0.507
HP_0039	HP_0040	85962.HP_0039	85962.HP_0040	0.773	0.862	0	0	0	0	0	0	0.967
HP_0039	HP_0527	85962.HP_0039	85962.HP_0527	0.063	0	0	0	0.642	0.895	0.182	0.096	0.969
HP_0040	HP_0530	85962.HP_0040	85962.HP_0530	0	0	0	0	0.472	0	0	0	0.472
HP_0040	HP_0525	85962.HP_0040	85962.HP_0525	0.042	0	0.518	0	0.476	0	0	0.045	0.738
HP_0040	HP_0524	85962.HP_0040	85962.HP_0524	0.047	0	0.768	0	0	0	0	0.045	0.77
HP_0040	cagE	85962.HP_0040	85962.HP_0544	0.043	0	0.765	0	0.449	0	0.141	0	0.879
HP_0040	HP_0459	85962.HP_0040	85962.HP_0459	0.043	0	0.775	0	0.449	0	0.141	0	0.884

HP_0040	HP_0527	85962.HP_0040	85962.HP_0527	0.063	0	0.761	0	0.642	0.895	0.182	0.096	0.992
HP_0459	HP_0530	85962.HP_0459	85962.HP_0530	0	0	0	0	0.405	0.318	0	0	0.576
HP_0459	HP_0528	85962.HP_0459	85962.HP_0528	0	0	0.362	0	0.449	0	0.141	0	0.671
HP_0459	HP_0524	85962.HP_0459	85962.HP_0524	0	0	0.775	0	0.053	0	0	0.041	0.777
HP_0459	HP_0525	85962.HP_0459	85962.HP_0525	0	0.001	0.728	0	0.346	0	0	0.041	0.814
HP_0459	HP_0527	85962.HP_0459	85962.HP_0527	0.084	0	0.775	0	0.596	0	0.167	0.276	0.94
HP_0524	cagT	85962.HP_0524	85962.HP_0532	0.352	0	0	0	0.046	0	0	0.563	0.706
HP_0524	HP_0530	85962.HP_0524	85962.HP_0530	0.387	0	0	0	0	0	0	0.586	0.735
HP_0524	cagE	85962.HP_0524	85962.HP_0544	0	0.058	0.775	0	0.053	0	0	0.508	0.888
HP_0524	HP_0528	85962.HP_0524	85962.HP_0528	0.568	0	0.388	0	0	0	0	0.636	0.895
HP_0524	HP_0525	85962.HP_0524	85962.HP_0525	0.774	0.161	0.727	0	0	0	0	0.541	0.973
HP_0524	HP_0527	85962.HP_0524	85962.HP_0527	0.604	0	0.775	0	0	0.282	0	0.631	0.973
HP_0525	cagT	85962.HP_0525	85962.HP_0532	0.216	0	0	0	0	0	0	0.507	0.597
HP_0525	HP_0530	85962.HP_0525	85962.HP_0530	0.387	0	0	0	0.363	0	0	0.611	0.835
HP_0525	HP_0528	85962.HP_0525	85962.HP_0528	0.472	0	0.229	0	0.476	0	0	0.639	0.913
HP_0525	cagE	85962.HP_0525	85962.HP_0544	0	0.222	0.751	0	0.346	0	0	0.461	0.922
HP_0525	HP_0527	85962.HP_0525	85962.HP_0527	0.505	0	0.731	0	0.495	0	0	0.625	0.971
HP_0527	HP_0530	85962.HP_0527	85962.HP_0530	0.594	0	0	0	0.609	0	0	0.61	0.933
HP_0527	cagE	85962.HP_0527	85962.HP_0544	0.084	0	0.775	0	0.596	0	0.167	0.558	0.963
HP_0527	cagT	85962.HP_0527	85962.HP_0532	0.289	0	0	0	0.044	0.8	0	0.767	0.964
HP_0527	HP_0528	85962.HP_0527	85962.HP_0528	0.751	0	0.671	0	0.642	0.978	0.182	0.814	0.999
HP_0528	cagE	85962.HP_0528	85962.HP_0544	0	0	0.502	0	0.449	0	0.141	0.534	0.875
HP_0528	HP_0530	85962.HP_0528	85962.HP_0530	0.613	0	0	0	0.472	0	0	0.591	0.909

HP_0528	cagT	85962.HP_0528	85962.HP_0532	0.447	0	0	0	0	0.8	0	0.638	0.956
HP_0530	cagT	85962.HP_0530	85962.HP_0532	0.285	0	0	0	0	0	0	0.526	0.646
HP_0530	cagE	85962.HP_0530	85962.HP_0544	0	0	0	0	0.405	0.318	0	0.439	0.752
cagE	cagT	85962.HP_0544	85962.HP_0532	0	0	0	0	0.192	0	0	0.726	0.769

Table S2.- Protein Interactions of CagY/Cag7 calculated by STRING server.

#term ID	term description	observed gene count	background gene count	strength	false discovery rate	matching proteins in your network (IDs)	matching proteins in your network (labels)
CL:2854	Mixed, incl. Bacterial secretion system, and Type IV secretion system, VirB10/TrbI	11	35	1.65	1.33E-15	85962.HP_0017,85962.HP_0039,85962.HP_0040,85962.HP_0459,85962.HP_0524,85962.HP_0525,85962.HP_0527,85962.HP_0528,85962.HP_0530,85962.HP_0532,85962.HP_0544	HP_0017,HP_0039,HP_0040,HP_0459,HP_0524,HP_0525,HP_0527,HP_0528,HP_0530,cagT,cagE
CL:2856	Bacterial secretion system, and Type IV secretion system, VirB10/TrbI	10	19	1.87	1.55E-15	85962.HP_0017,85962.HP_0039,85962.HP_0040,85962.HP_0524,85962.HP_0525,85962.HP_0527,85962.HP_0528,85962.HP_0530,85962.HP_0532,85962.HP_0544	HP_0017,HP_0039,HP_0040,HP_0524,HP_0525,HP_0527,HP_0528,HP_0530,cagT,cagE
CL:2859	Bacterial secretion system, and Type IV secretion system, VirB10/TrbI	7	10	2	8.00E-11	85962.HP_0524,85962.HP_0525,85962.HP_0527,85962.HP_0528,85962.HP_0530,85962.HP_0532,85962.HP_0544	HP_0524,HP_0525,HP_0527,HP_0528,HP_0530,cagT,cagE
CL:2869	Mixed, incl. Protein-exporting ATPase activity, and Type II/IV secretion system protein	4	5	2.06	8.25E-06	85962.HP_0524,85962.HP_0525,85962.HP_0530,85962.HP_0544	HP_0524,HP_0525,HP_0530,cagE
CL:2860	Mixed, incl. Conjugal transfer protein, and Type IV secretion system, VirB10/TrbI	3	5	1.93	0.00063	85962.HP_0527,85962.HP_0528,85962.HP_0532	HP_0527,HP_0528,cagT
CL:2881	Mixed, incl. Conjugal transfer protein, and Helicase HerA, central domain	3	5	1.93	0.00063	85962.HP_0017,85962.HP_0039,85962.HP_0040	HP_0017,HP_0039,HP_0040

Table S3.- Protein Templates used by the I-TASSER server to model the MRR of CagY.

PDB Hit	PDB accesion	RMSD	Protein name
1	7kogB	0.675	Lethocerus Myosin II complete coiled-coil domain resolved in its native environment
2	3hr2A	0.256	Low resolution, molecular envelope structure of type I collagen in situ determined by fiber diffraction. Single type I collagen molecule, post rigid body refinement, 'relaxed'
3	1c1gA	0.193	CRYSTAL STRUCTURE OF TROPOMYOSIN AT 7 ANGSTROMS RESOLUTION IN THE SPERMINE-INDUCED CRYSTAL FORM
4	6yvuB	0.171	Condensin complex from S.cerevisiae ATP-free apo non-engaged state
5	6f1tX	0.168	Cryo-EM structure of two dynein tail domains bound to dynactin and BICDR1
6	6z9lA	0.163	Enterococcal PrgA
7	1sjjA	0.161	Cryo-EM Structure of Chicken Gizzard Smooth Muscle alpha-Actinin
8	6jlbA	0.158	Crystal structure of lamin A/C fragment and assembly mechanisms of intermediate filaments
9	6l5jA	0.155	Crystal structure of human rootletin 1108-1317

Table S4.- Predicted disulfide bonds by PDBsum.

1st cysteine	2nd cysteine	Type (*)	Chi1	Chi2	Chi3	Chi2p	Chi1p
A 92	A 102	LHS	-179.7	-88.4	-73.6	-53.6	-64.9
A 125	A 138	LHS	-177.3	-65.0	-77.7	-66.9	-64.7
A 163	A 176	LHS	-178.5	-61.7	-65.8	-67.2	-65.6
A 205	A 218	LHS	-176.6	-67.7	-78.3	-62.3	-61.1
A 237	A 250	LHS	-177.0	-67.7	-73.0	-60.8	-63.2
A 274	A 287	LHS	-179.3	-79.1	-71.6	-67.7	-61.9
A 306	A 319	LHS	-178.0	-71.8	-68.0	-85.2	-61.7
A 343	A 356	LHS	-179.6	-76.7	-69.1	-71.9	-65.4
A 382	A 395	LHS	179.1	-75.8	-73.5	-63.5	-63.7
A 420	A 433	LHS	-179.4	-85.1	-64.9	-78.1	-63.2
A 452	A 465	LHS	-176.6	-79.1	-62.5	-79.9	-61.0
A 489	A 502	LHS	179.8	-78.8	-72.3	-63.8	-63.1
A 527	A 540	LHS	-178.6	-65.4	-73.7	-71.1	-63.5
A 566	A 579	LHS	-179.9	-76.8	-76.5	-55.8	-62.3
A 604	A 617	LHS	-179.0	-74.9	-71.5	-67.5	-62.8
A 636	A 649	LHS	-177.4	-70.3	-69.2	-77.4	-61.7
A 673	A 686	LHS	-178.5	-64.1	-73.7	-71.6	-62.9
A 711	A 724	LHS	179.9	-76.8	-72.5	-64.7	-62.9
A 749	A 762	LHS	-178.9	-72.8	-71.2	-77.2	-61.6
A 781	A 794	LHS	-178.3	-83.7	-61.3	-88.6	-62.7
A 818	A 831	LHS	-179.9	-80.4	-66.3	-77.8	-63.7
A 856	A 869	LHS	-177.8	-78.5	-71.6	-61.9	-62.8
A 895	A 908	LHS	-177.8	-81.1	-63.3	-81.3	-63.2
A 927	A 940	LHS	-177.9	-75.0	-63.7	-91.0	-63.9
A 964	A 977	LHS	-178.5	-66.0	-75.7	-74.1	-62.3
A 1003	A 1016	LHS	-179.5	-76.8	-75.1	-59.6	-63.6
A 1042	A 1057	LHS	-174.6	-87.2	-59.0	-79.0	-63.3
A 1081	A 1094	LHS	-175.2	-84.9	-53.6	-78.8	-66.7

(*) The data displayed for each disulphide bond is as follows: The table shows the residue numbers of the two cysteines involved in the disulphide bridge and the type of disulphide. The type of bridge is abbreviated (RHH: right hand hook; SRH: short right hand hook; LHS: left handed spiral; RHS: right handed spiral). Chi1, chi2, chi3, chi2' and chi1' values and the distance between the C-alpha atoms of the residues involved are also recorded.

Table S5.- AP sequences

AP model	Sequence
WT	FLQTAPIIALDKLIGLGKGRSERTPEFNYALGQAINGSMQSSAQMSNQILGQ
GS20	FLQTAPIIALDKLIGLGSGSGSGSGSGSGSGSGSGSGSMQSSAQMSNQILGQ
Xc (*)	FLQTAPIIALDKLIGVGSGIVTQQPFESNTSMQSSAQMSNQILGQ

(*) Xanthomonas citri
