# Supplementary figures and images for: A Proposal for a Consolidated Structural Model of the CagY Protein of Helicobacter pylori
Source: Int J Mol Sci. 2023 Nov 26;24(23):16781. doi: 10.3390/ijms242316781 (PMC10706595; doi:10.3390/ijms242316781)

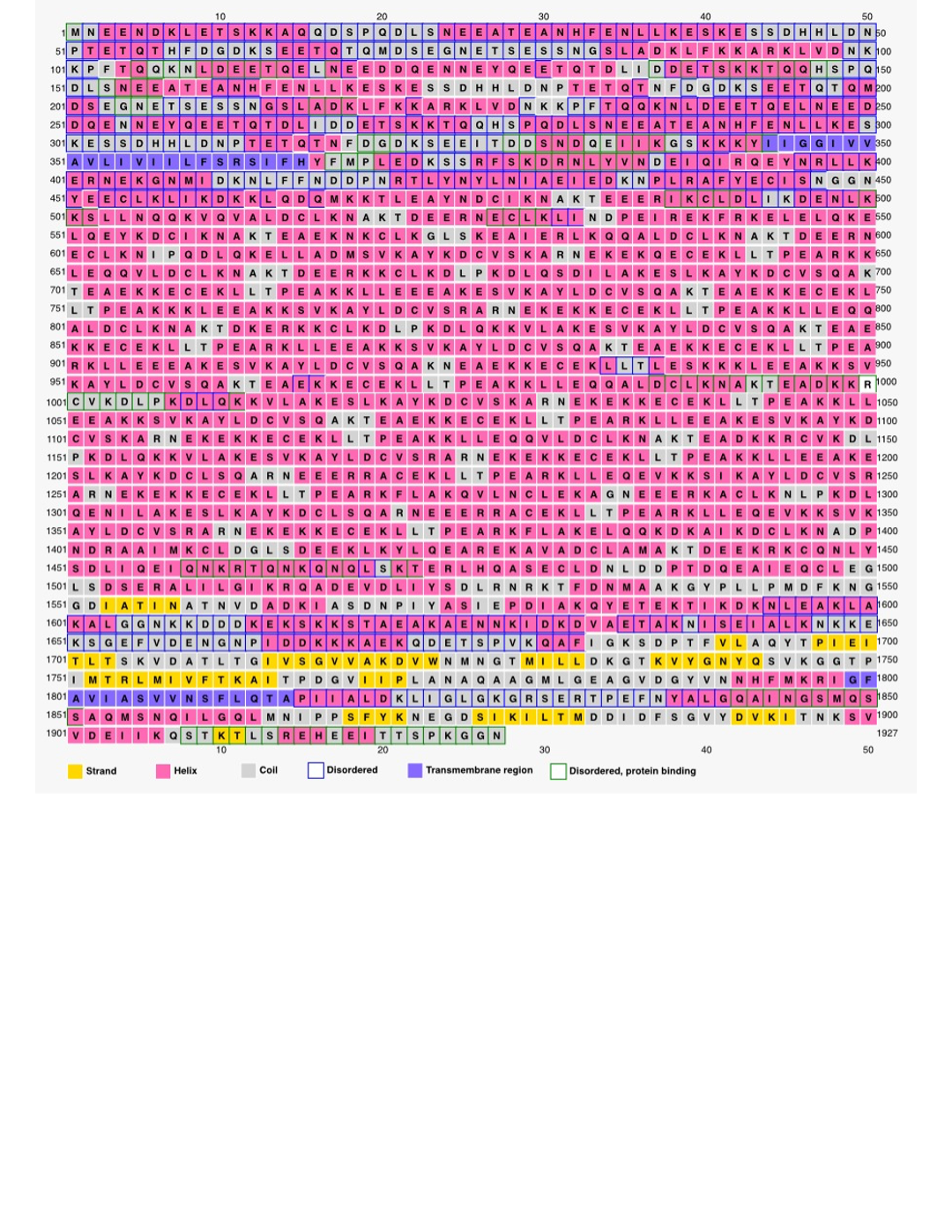

Supplement: Supplementary file 1 [file ijms-24-16781-s001.zip › Supplementary_material/Figures/FigS1.png]

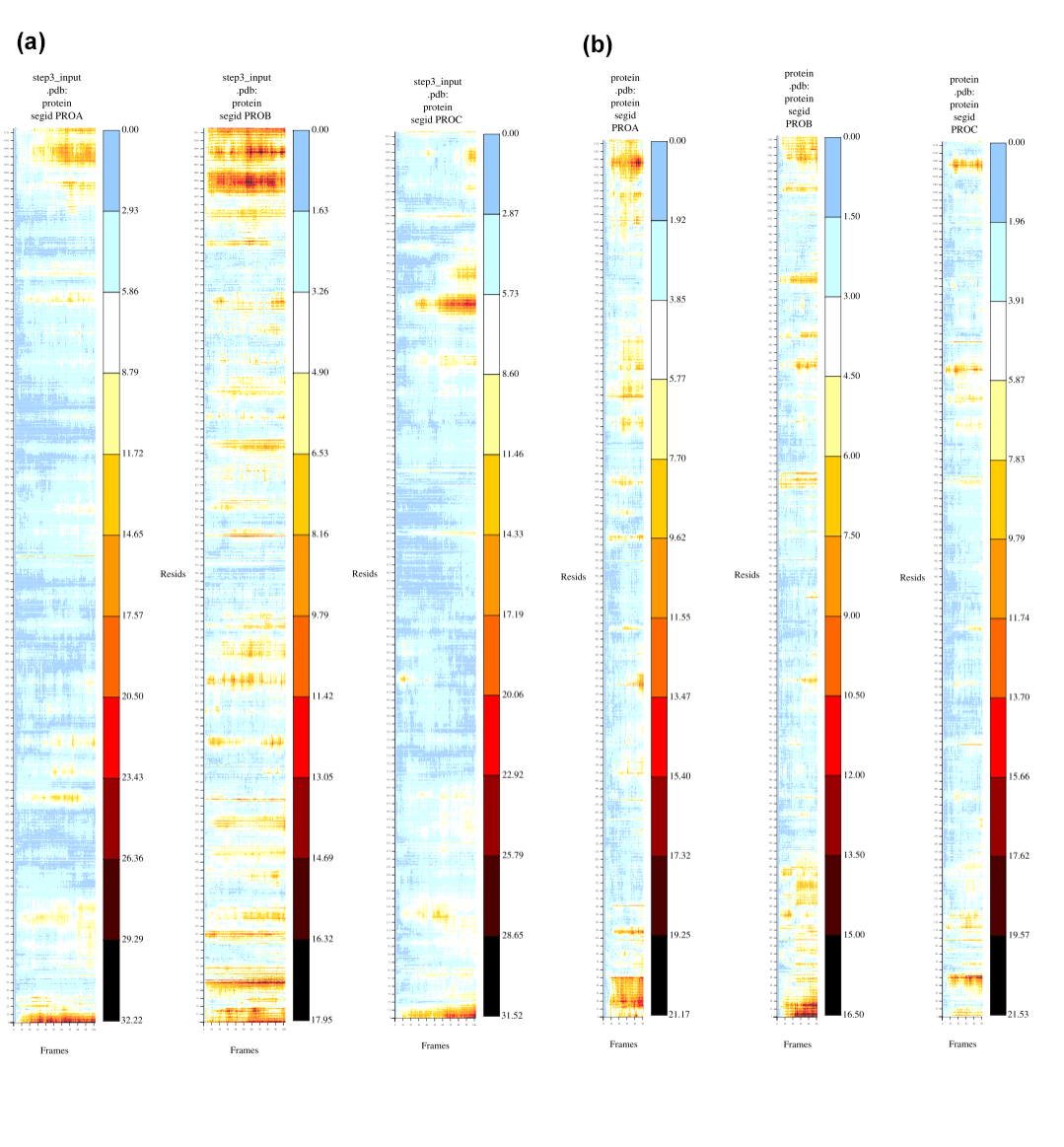

Supplement: Supplementary file 1 [file ijms-24-16781-s001.zip › Supplementary_material/Figures/FigS10.png]

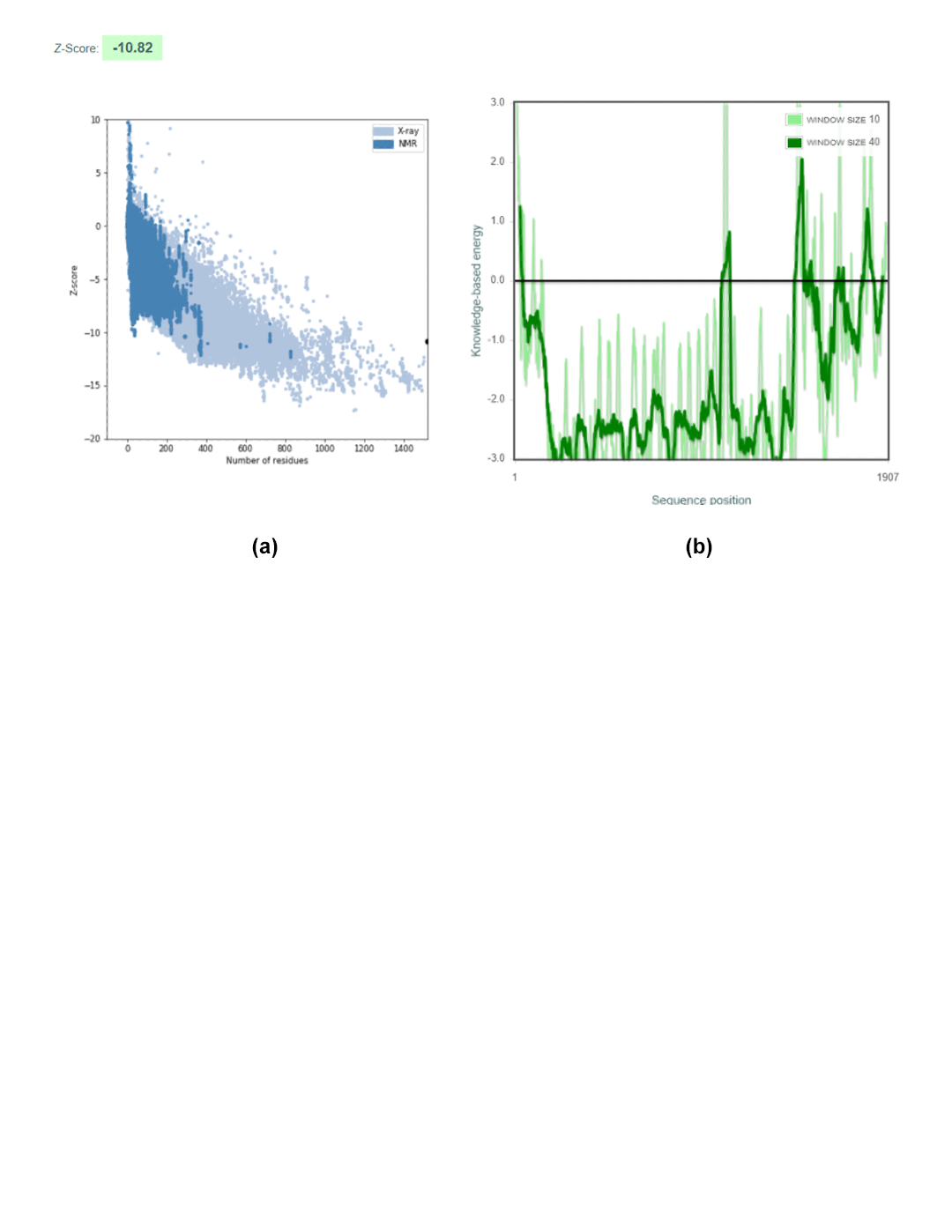

Supplement: Supplementary file 1 [file ijms-24-16781-s001.zip › Supplementary_material/Figures/FigS11.png]

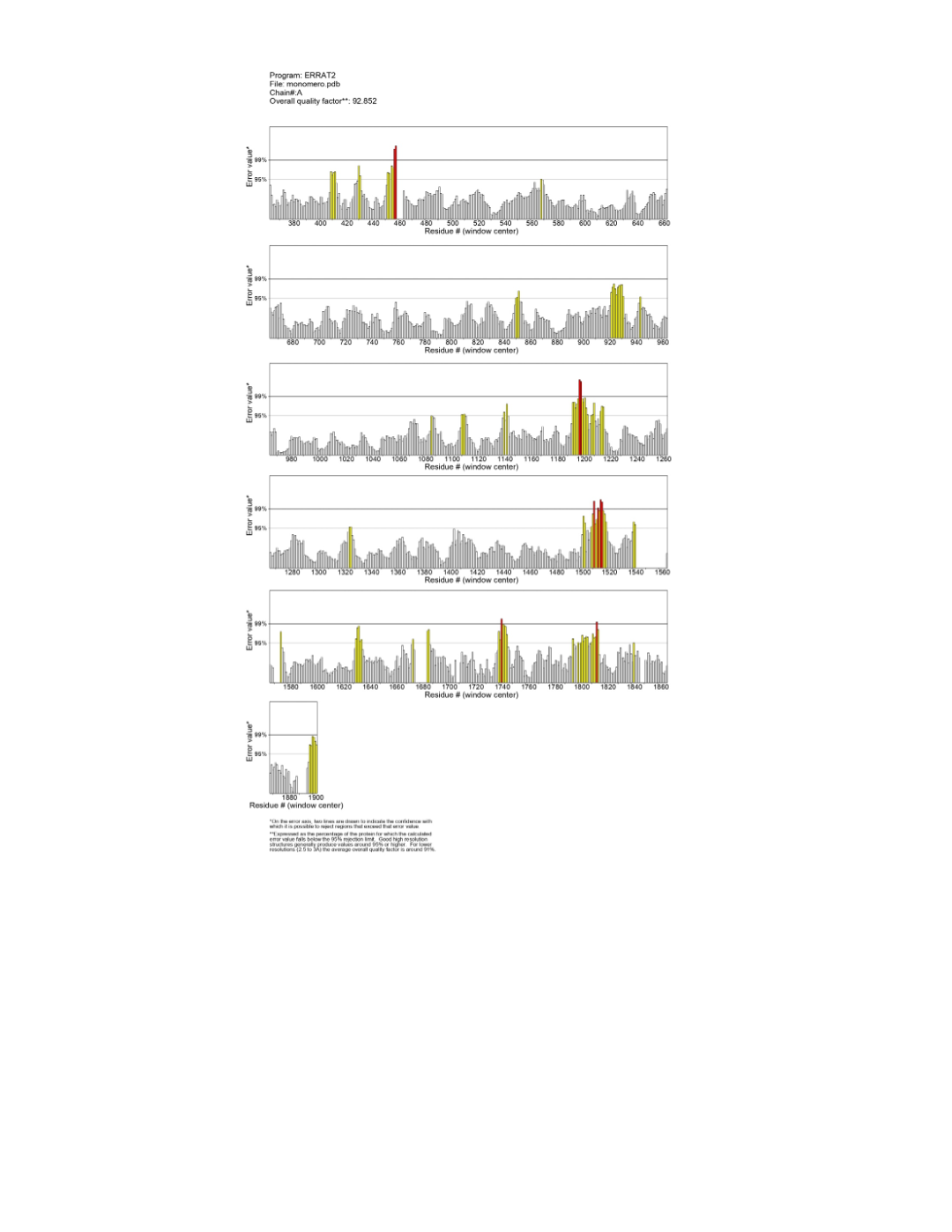

Supplement: Supplementary file 1 [file ijms-24-16781-s001.zip › Supplementary_material/Figures/FigS12.png]

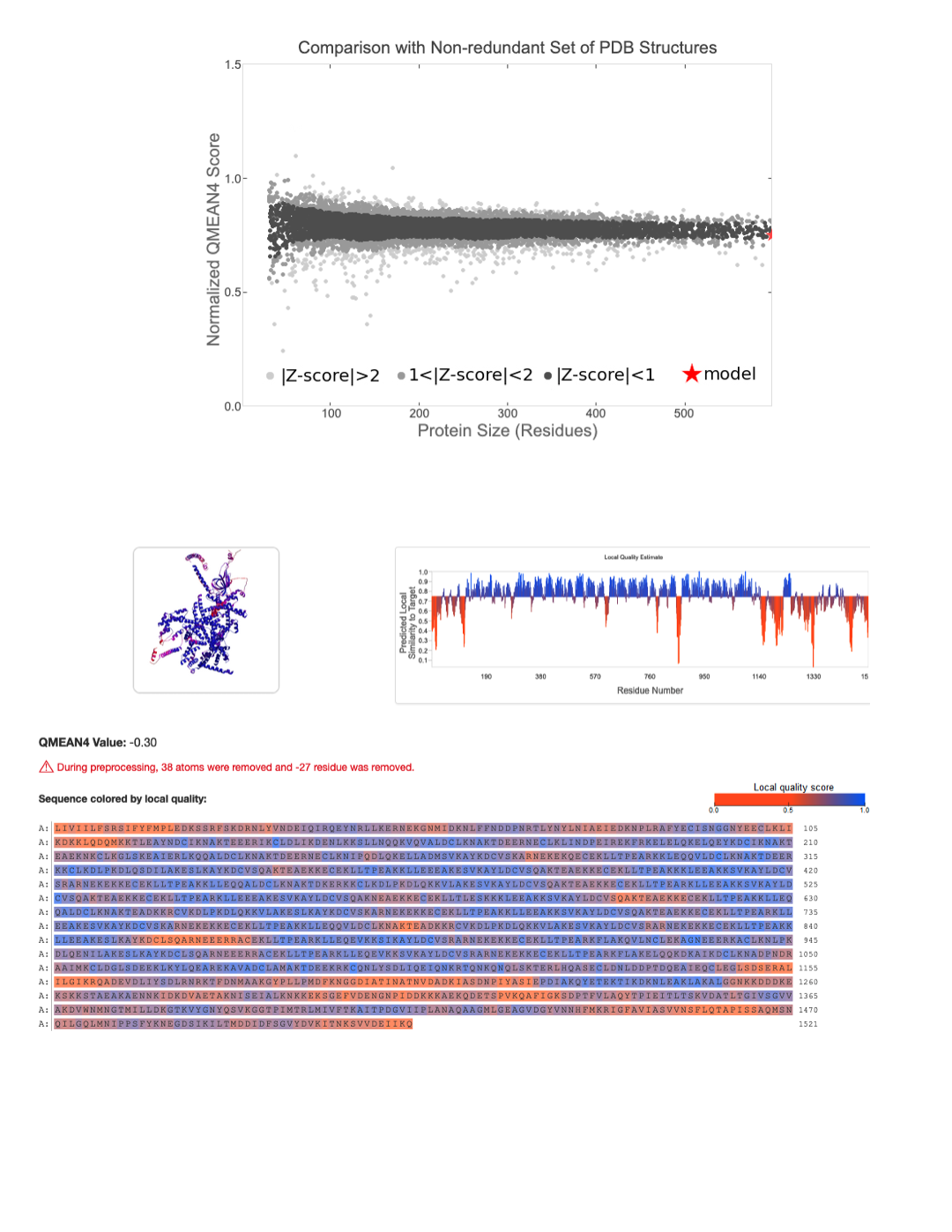

Supplement: Supplementary file 1 [file ijms-24-16781-s001.zip › Supplementary_material/Figures/FigS13.png]

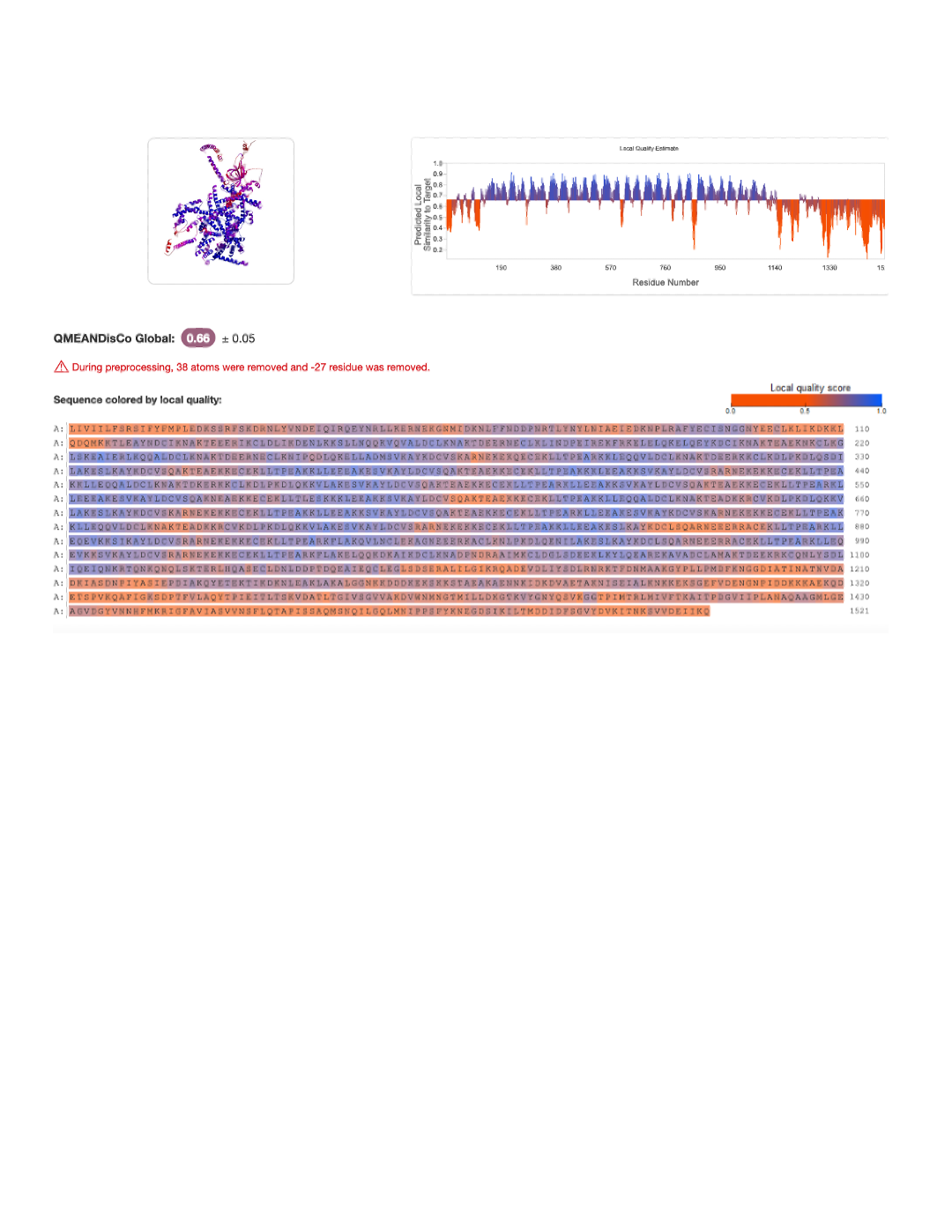

Supplement: Supplementary file 1 [file ijms-24-16781-s001.zip › Supplementary_material/Figures/FigS14.png]

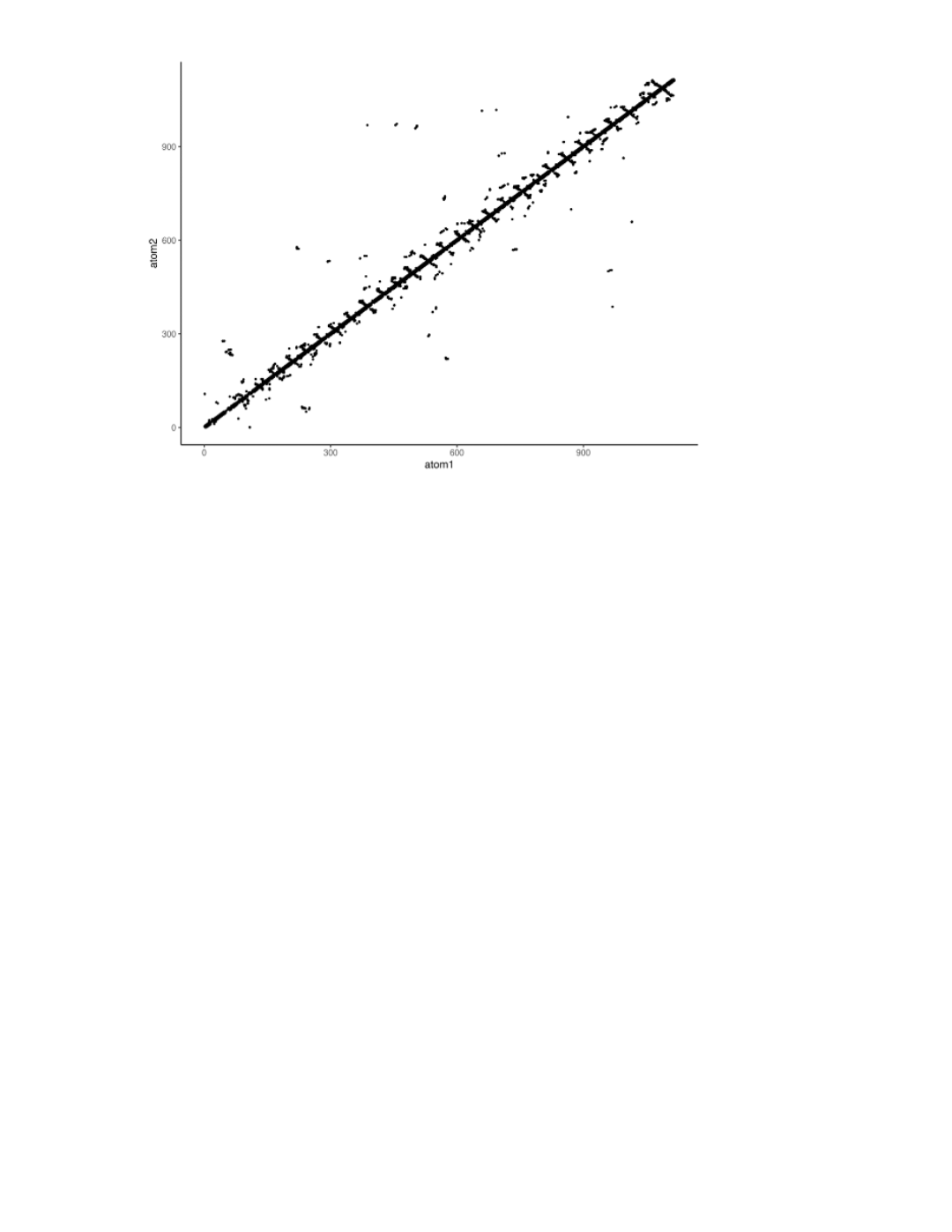

Supplement: Supplementary file 1 [file ijms-24-16781-s001.zip › Supplementary_material/Figures/FigS15.png]

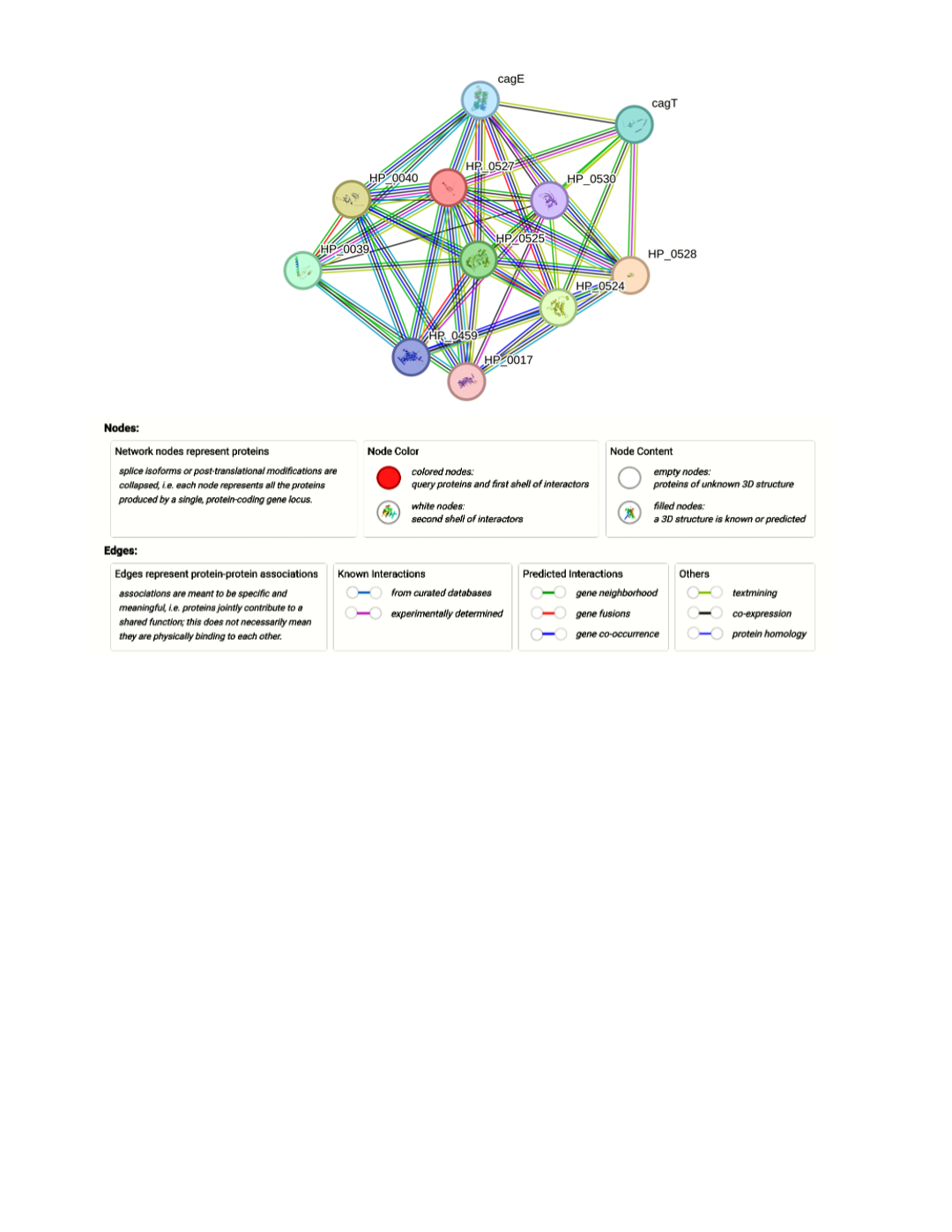

Supplement: Supplementary file 1 [file ijms-24-16781-s001.zip › Supplementary_material/Figures/FigS16.png]

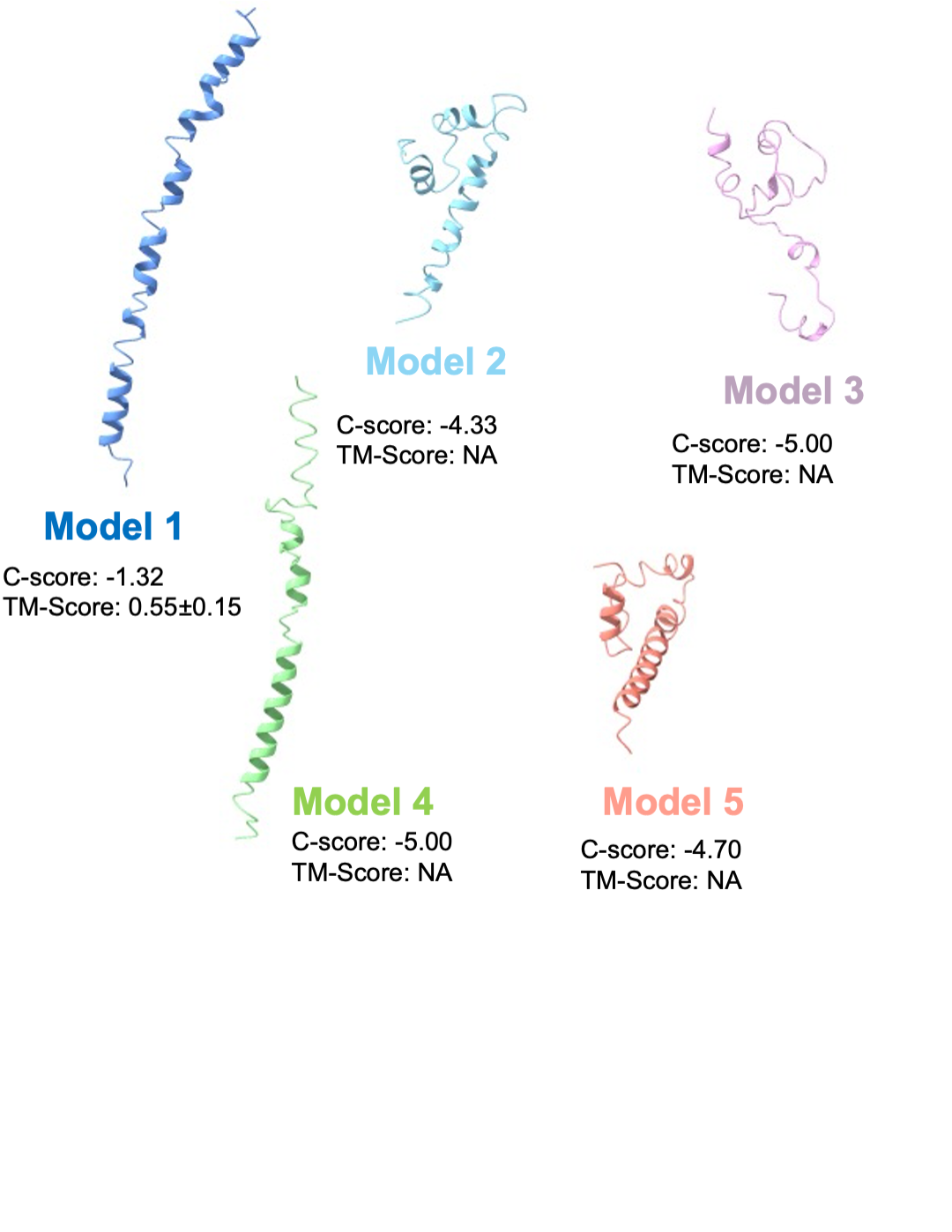

Supplement: Supplementary file 1 [file ijms-24-16781-s001.zip › Supplementary_material/Figures/FigS2.png]

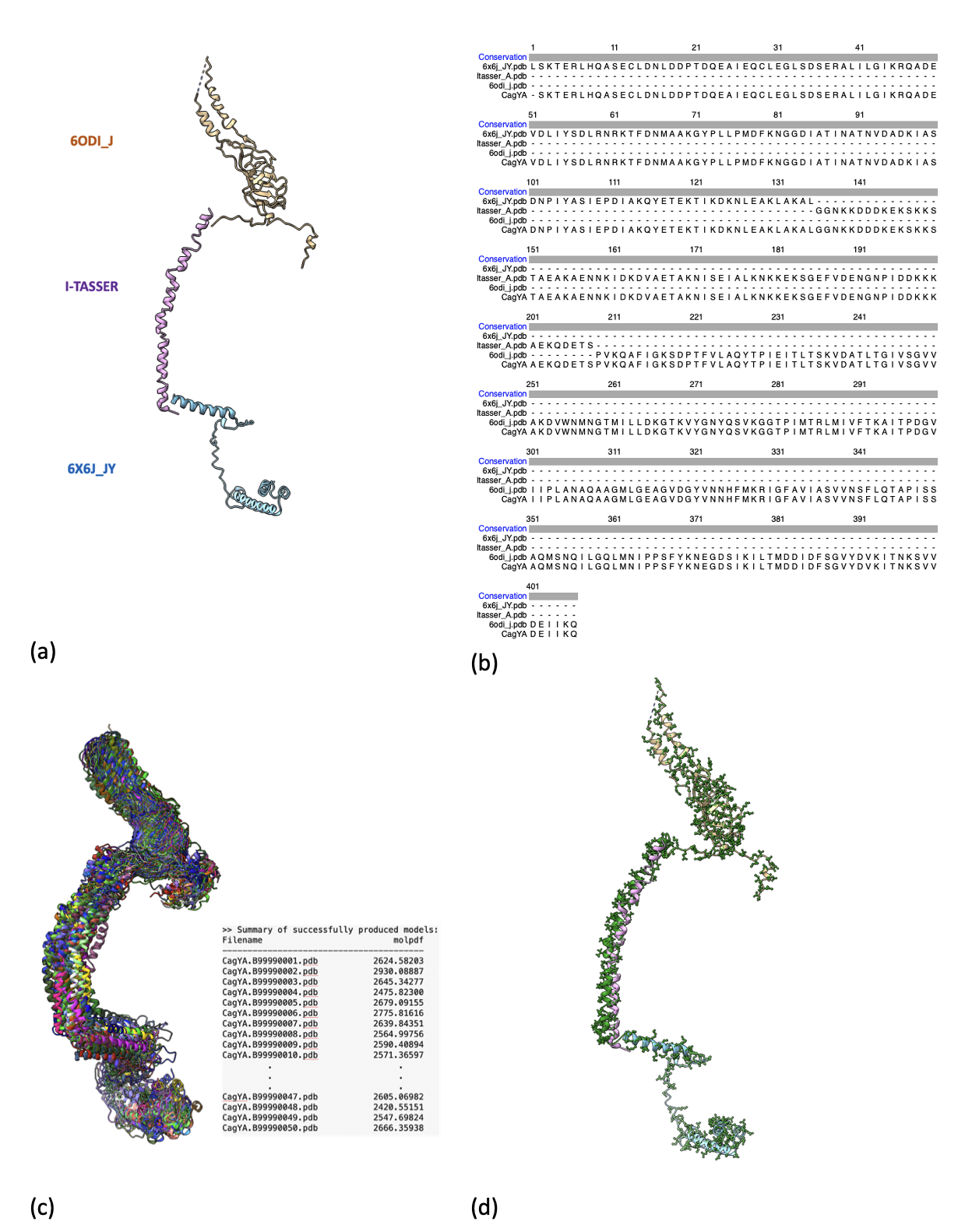

Supplement: Supplementary file 1 [file ijms-24-16781-s001.zip › Supplementary_material/Figures/FigS3.png]

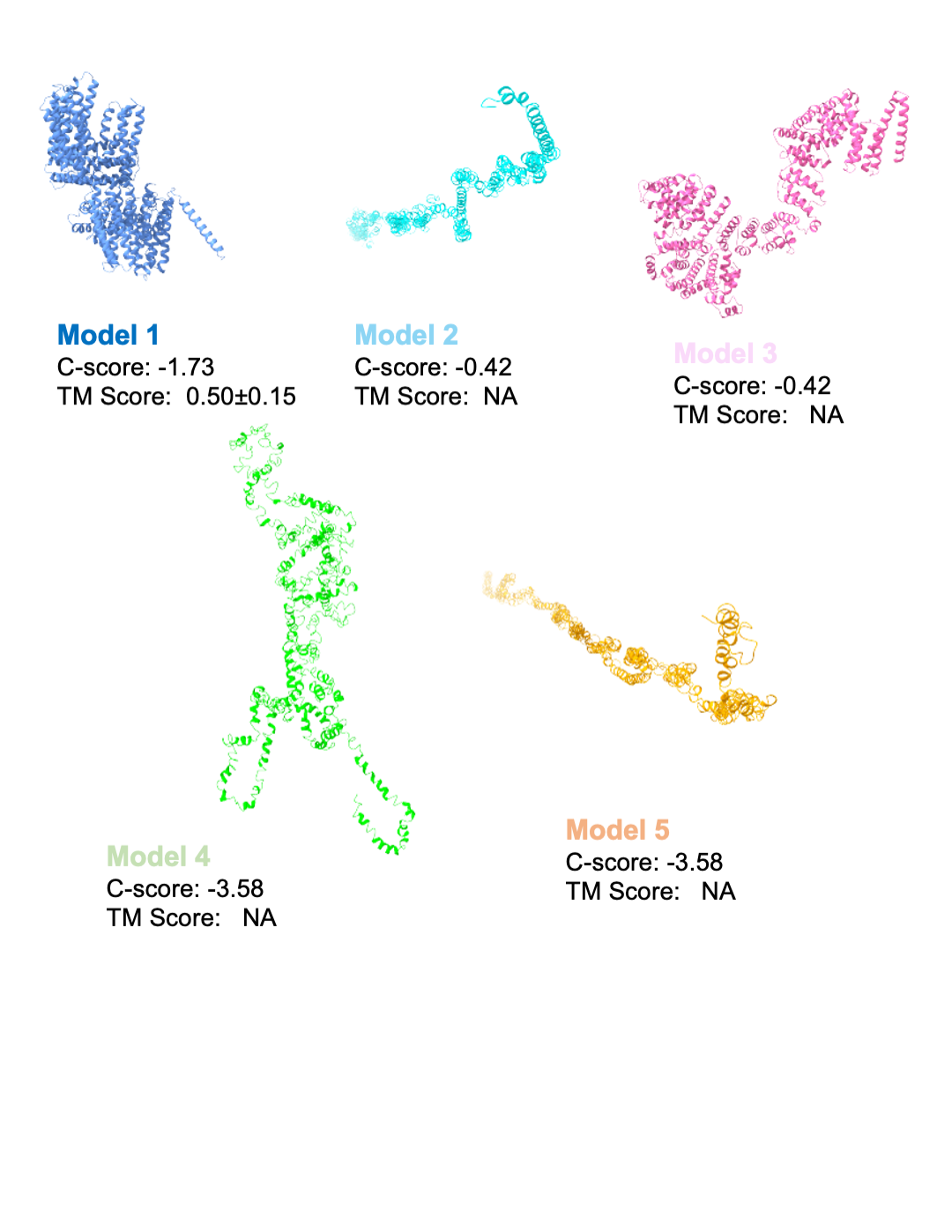

Supplement: Supplementary file 1 [file ijms-24-16781-s001.zip › Supplementary_material/Figures/FigS4.png]

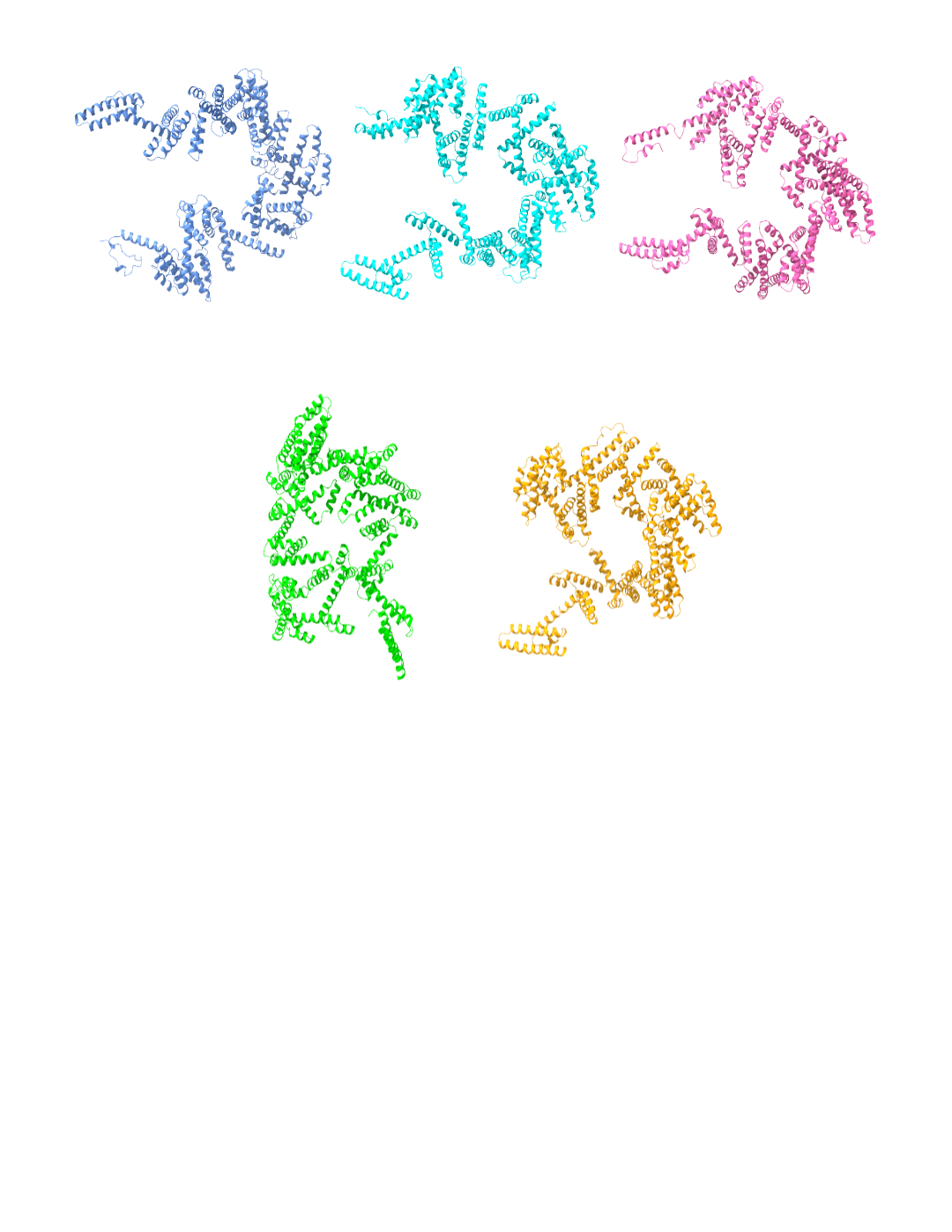

Supplement: Supplementary file 1 [file ijms-24-16781-s001.zip › Supplementary_material/Figures/FigS5.png]

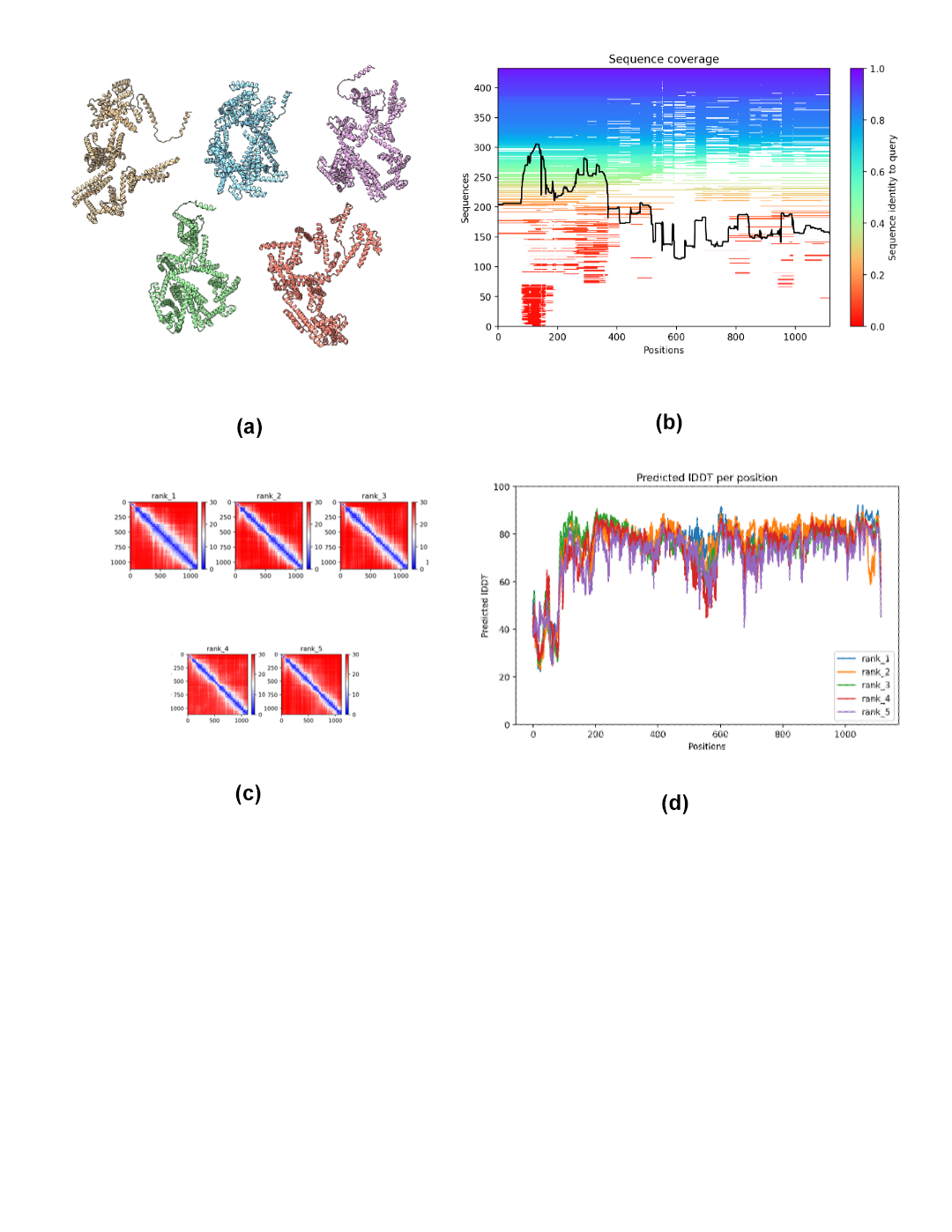

Supplement: Supplementary file 1 [file ijms-24-16781-s001.zip › Supplementary_material/Figures/FigS6.png]

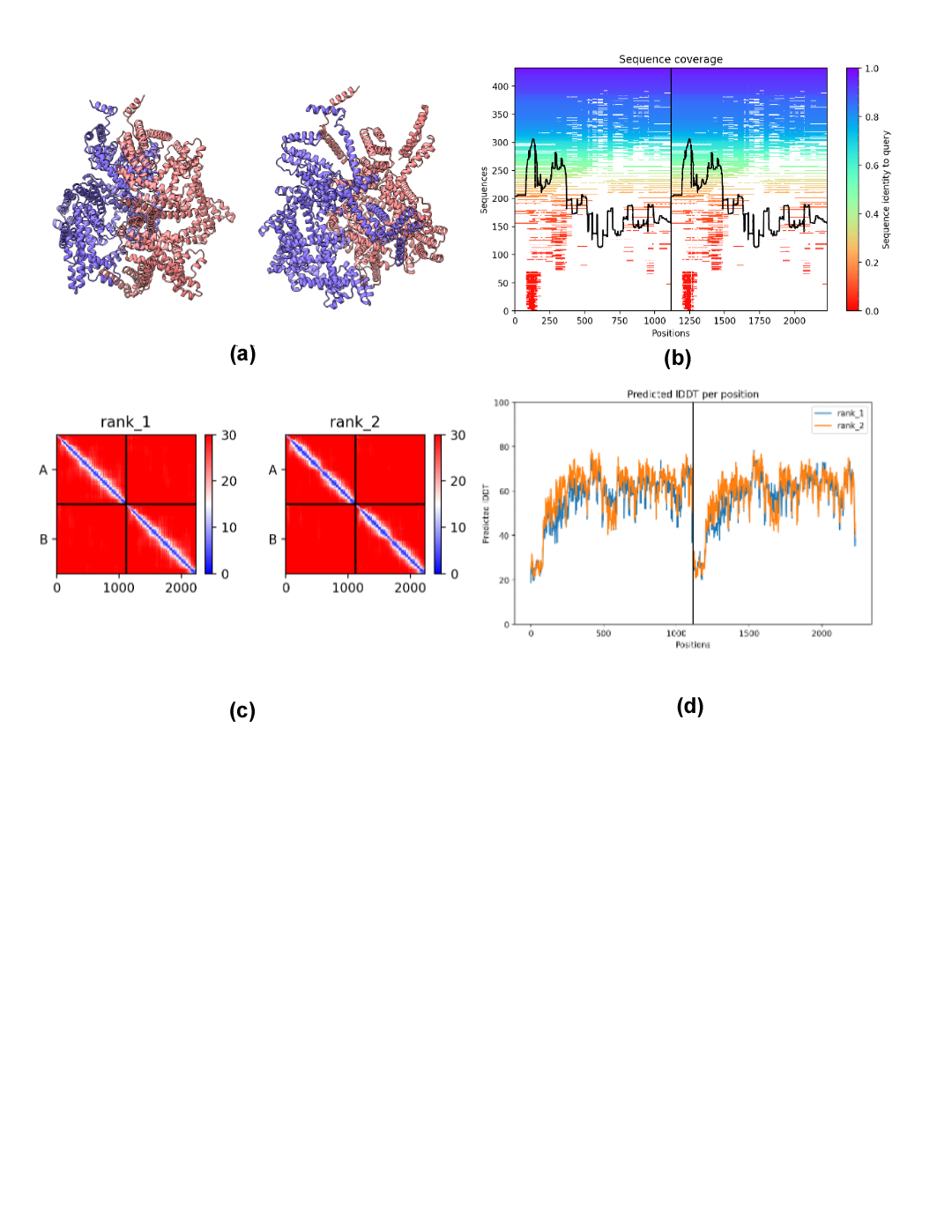

Supplement: Supplementary file 1 [file ijms-24-16781-s001.zip › Supplementary_material/Figures/FigS7.png]

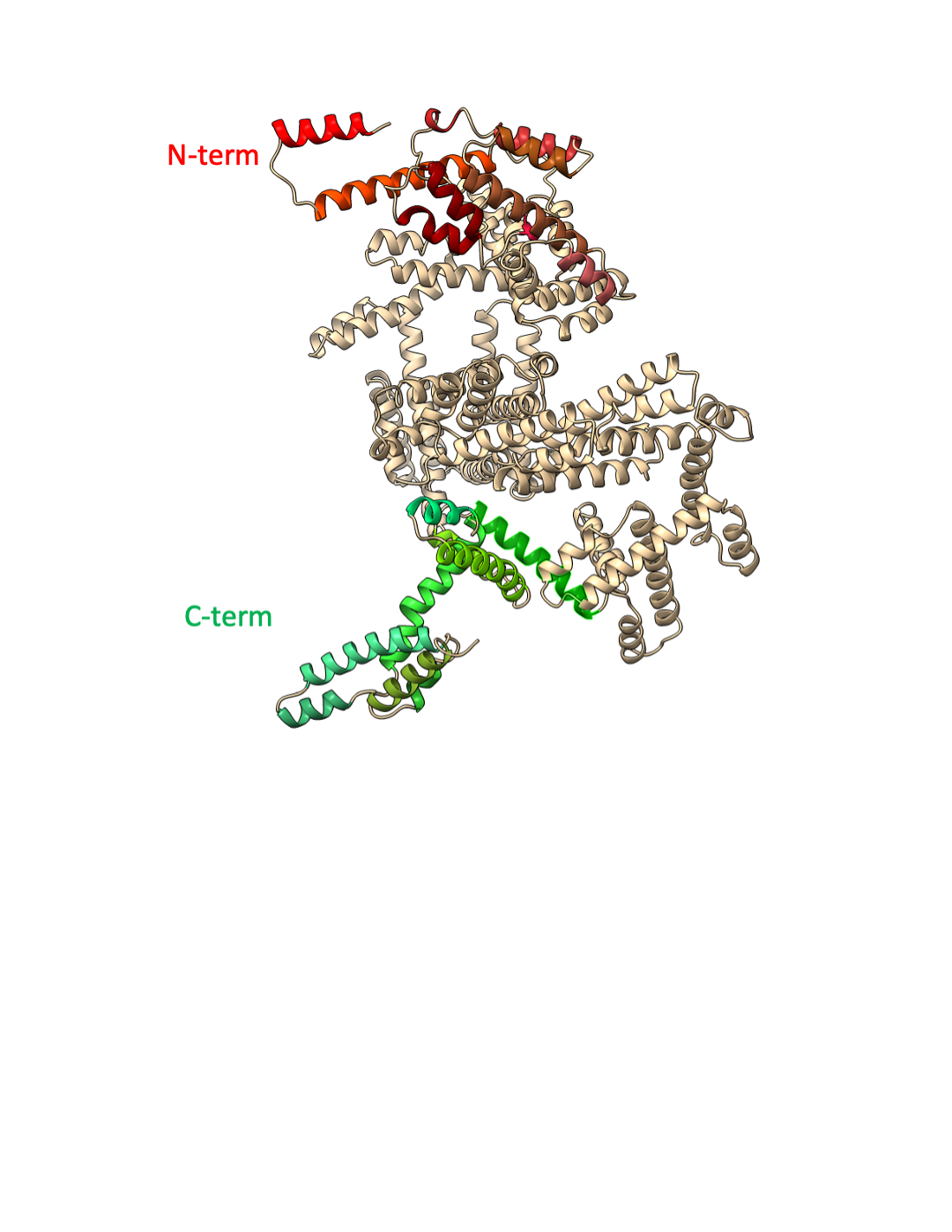

Supplement: Supplementary file 1 [file ijms-24-16781-s001.zip › Supplementary_material/Figures/FigS8.png]

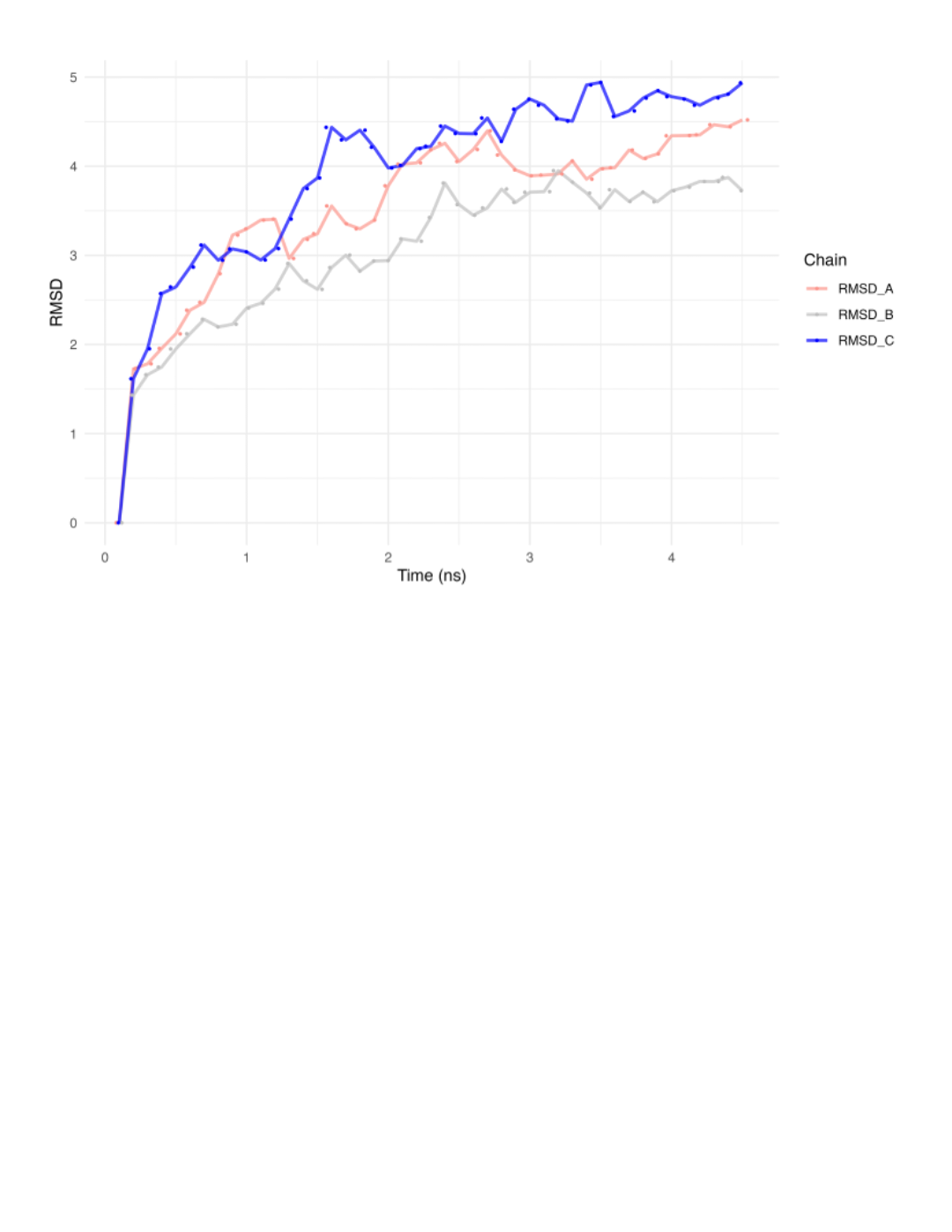

Supplement: Supplementary file 1 [file ijms-24-16781-s001.zip › Supplementary_material/Figures/FigS9.png]
